# An Atlas of Peroxiredoxins Created Using an Active Site Profile-Based Approach to Functionally Relevant Clustering of Proteins

**DOI:** 10.1371/journal.pcbi.1005284

**Published:** 2017-02-10

**Authors:** Angela F. Harper, Janelle B. Leuthaeuser, Patricia C. Babbitt, John H. Morris, Thomas E. Ferrin, Leslie B. Poole, Jacquelyn S. Fetrow

**Affiliations:** 1 Department of Physics, Wake Forest University, Winston-Salem, North Carolina, United States of America; 2 Department of Molecular Genetics and Genomics, Wake Forest School of Medicine, Winston-Salem, North Carolina, United States of America; 3 Department of Bioengineering and Therapeutic Sciences, University of California San Francisco School of Pharmacy, San Francisco, California, United States of America; 4 Department of Pharmaceutical Chemistry, University of California San Francisco School of Pharmacy, San Francisco, California, United States of America; 5 Department of Biochemistry, Wake Forest School of Medicine, Winston-Salem, North Carolina, United States of America; 6 Department of Chemistry, University of Richmond, Richmond, Virginia, United States of America; University College London, UNITED KINGDOM

## Abstract

Peroxiredoxins (Prxs or Prdxs) are a large protein superfamily of antioxidant enzymes that rapidly detoxify damaging peroxides and/or affect signal transduction and, thus, have roles in proliferation, differentiation, and apoptosis. Prx superfamily members are widespread across phylogeny and multiple methods have been developed to classify them. Here we present an updated atlas of the Prx superfamily identified using a novel method called MISST (Multi-level Iterative Sequence Searching Technique). MISST is an iterative search process developed to be both agglomerative, to add sequences containing similar functional site features, and divisive, to split groups when functional site features suggest distinct functionally-relevant clusters. Superfamily members need not be identified initially—MISST begins with a minimal representative set of known structures and searches GenBank iteratively. Further, the method’s novelty lies in the manner in which isofunctional groups are selected; rather than use a single or shifting threshold to identify clusters, the groups are deemed isofunctional when they pass a self-identification criterion, such that the group identifies itself and nothing else in a search of GenBank. The method was preliminarily validated on the Prxs, as the Prxs presented challenges of both agglomeration and division. For example, previous sequence analysis clustered the Prx functional families Prx1 and Prx6 into one group. Subsequent expert analysis clearly identified Prx6 as a distinct functionally relevant group. The MISST process distinguishes these two closely related, though functionally distinct, families. Through MISST search iterations, over 38,000 Prx sequences were identified, which the method divided into six isofunctional clusters, consistent with previous expert analysis. The results represent the most complete computational functional analysis of proteins comprising the Prx superfamily. The feasibility of this novel method is demonstrated by the Prx superfamily results, laying the foundation for potential functionally relevant clustering of the universe of protein sequences.

## Author Summary

Peroxiredoxins (Prxs) are a large, ubiquitous superfamily of proteins that are arguably the most important reductants of peroxide in biological systems. These proteins are involved in a diverse array of essential cellular functions, including peroxide reduction, signal transduction, circadian rhythms, chaperone function and apoptosis. Previously, Prxs have been classified multiple ways, based on biological role and evolutionary analysis. A more detailed expertly curated analysis identified six functionally relevant Prx classes and identified over 3500 proteins in these six classes; this set provides a validation for molecular function annotation methods. It is well-known that automated molecular functional annotation for individual protein sequences is difficult without detailed manual curation. In this work, we address this deficiency in available technologies by presenting a novel iterative method, MISST, for agglomeratively identifying superfamily members and clustering them into functionally relevant groups. Using this potentially automatable approach, 38,739 Prx sequences were identified from GenBank. MISST identified six functionally relevant clusters from these sequences, matching those previously identified by experts. Key mechanistic determinants and organismal distribution are explored. This analysis provides a significantly more complete understanding of this biologically important protein superfamily; the method lays a foundation for automated functionally relevant clustering of the protein universe.

## Introduction

Peroxiredoxins (Prxs) are a large and ubiquitous superfamily of thiol dependent peroxidases, which have long been known to be involved in the reduction of aliphatic and aromatic hydroperoxides and peroxynitrite in biological systems [1–3]. Historically, these proteins have also been called TSA (thiol-specific antioxidant), AhpC (alkyl hydroperoxide reductase), and TPx (thioredoxin peroxidase). Prxs are known to protect cellular components from oxidative damage [4,5]. Indeed, it has been argued that Prxs are one of the most important peroxide scavengers in biological systems [6–9].

In addition to a peroxide scavenger role, Prxs are involved in essential biological processes such as redox signaling, which, because of the Prx reaction efficiency, can occur by one of two mechanisms. In the first mechanism, oxidation of redox-regulated proteins is not caused by H_2_O_2_ directly, but is rather mediated by Prxs, such that Prx C_P_ is first oxidized by H_2_O_2_, which then reacts directly with the regulated kinase or phosphatase modifying its function. The regulated protein is subsequently regenerated by a cellular reductant. This signal transduction mechanism has been extensively reviewed [10–12]. In the second signaling mechanism, redox-regulated proteins may be directly oxidized by H_2_O_2_ [11,13–16]. However, thiol oxidation by H_2_O_2_ in redox regulated proteins is typically much slower in cellular proteins than the corresponding H_2_O_2_ detoxification by Prxs [17]. Thus, signal propagation occurs by Prx inactivation: Prxs are subject to H_2_O_2_ hyperoxidation at the active site cysteine, peroxidatic Cys (C_P_), which inactivates them (until they are repaired by the enzyme sulfiredoxin) [18,19]. The “floodgate hypothesis” posits that localized Prx inactivation (e.g. via hyperoxidation) serves to promote H_2_O_2_-mediated oxidation of redox-regulated proteins [20] and examples of such signaling in cells are emerging [21,22]. Hyperoxidation is also reported to play a role in circadian rhythms [23] and chaperone function [24]. Fine control of the Prx reaction mechanism is clearly essential; thus, understanding molecular function of this large and complex superfamily would provide insight into broader biological mechanisms.

As one would expect, peroxide detoxification and redox regulatory systems can be quite complex. For example, mammalian cells express six Prx isoforms: 2-Cys (PrxI, PrxII, PrxIII, and PrxIV), atypical 2-Cys (PrxV), and 1-Cys (PrxVI) [25]. Chloroplasts contain three Prx isoforms [26]. All Prxs contain C_P_ preceded in the sequence by a conserved **P**xxx**(T/S)**xx**C**_**P**_, a definitive motif for the Prx superfamily. An Arg is also absolutely conserved, but is contributed by a sequence fragment close in structure and distant in sequence. These residues activate the peroxide substrate, catalyze peroxide bond breakage, and catalyze attack of the C_P_ thiolate on the substrate hydroxyl [27–29].

The extent and importance of the Prx proteins has led to several approaches to cluster the superfamily based on active site details. At its most simple, Prxs are classified into typical 2-Cys, atypical 2-Cys, and 1-Cys Prx families based on the presence or absence and position of a resolving Cys, C_R_ [30,31]; however, proteins may have structural features of one of these classes, but mechanistic details of another [32]. Detailed sequence comparison and evolutionary analysis determined that Prxs diverged from an ancestor of the thioredoxin fold family and identified four classes of Prx, which these researchers called Prx1, Prx2, Prx3, and Prx4 [33]. Subsequent work based on detailed sequence analysis divided the Prx superfamily into six isofunctional families: AhpC/Prx1 (abbreviated Prx1), Prx6, Prx5, Tpx (thiol peroxidase), PrxQ/BCP (bacterioferritin comigratory protein, abbreviated PrxQ) and AhpE [28,32,34]. This level of detailed molecular functional annotation is typically lacking in the sequence databases, as we have previously shown [35].

**Fig 1:**
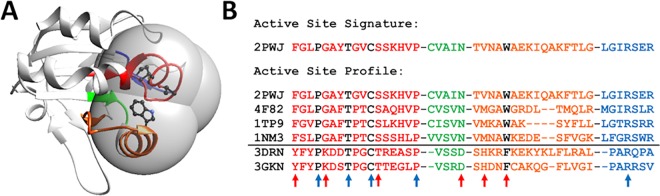
Active Site Profiling identifies molecular features around a protein’s functional site. (A) In an enzyme structure, key functional residues (black side chains) are identified from sequence and structural analysis. All residues within 10 Å of any key residue (gray spheres) are identified [35,36]. The visualization was created using UCSF Chimera package, version 1.10.2 (B) Residues within the 10 Å spheres are extracted and concatenated to form an active site signature (top). Signatures from a protein family are aligned to create an active site profile (ASP) (bottom). Within the profile, molecular features that are common across the superfamily (blue arrows), as well as features that seem to divide the profile into two distinct groups (red arrows), can be identified. The black line separates the two functional families with Prx5 proteins on top of the line and PrxQ proteins below the line.

More recently, we have used a bioinformatics approach based on active site profiling [36] to identify sequences in a given isofunctional family based on active site features [35,37]. Active site profiles (ASPs, Fig 1) are used to identify and compare functional site features. The Deacon Active Site Profiler (DASP), a tool that uses ASPs to search databases for sequences containing active site features similar to those in the ASP [37,38] identified many additional Prx members of each expertly identified isofunctional group [35]. Using this single search approach, we identified over 3500 proteins in the six Prx functional subgroups; these sequences are available in the Prx database, PREX [39] and in the Structure Function Linkage Database, SFLD [40,41]. SFLD curators subsequently added sequences to these groups using their hidden Markov model (HMM) approach.

The significant question is: could one automatically identify such isofunctional families within a protein superfamily without expert analysis? Databases such as CATH [42,43], PFAM [44,45] and SCOP [46,47] have clustered large superfamilies of proteins based upon domain characteristics and/or structural and sequence classification. Such approaches capture broad levels of functional similarity. On the other hand, in SFLD, proteins are clustered based on functional similarity [41]. An SFLD superfamily contains proteins that share part (but not all) of their enzyme mechanism. At a more detailed level, SFLD families contain proteins which exhibit the same enzyme mechanism (i.e., are isofunctional). CATH PFAM, and SCOP families are more similar to what is defined as an SFLD superfamily [40]; such broad groups usually contain multiple isofunctional families. Our goal is to develop a method to more automatically identify isofunctional clusters.

Several approaches aim to cluster sequences into isofunctional clusters, including FunFHMMer [48] (an updated version of GeMMA [49]), SCI-PHY [50], and ASMC [51]. These methods start with known superfamily sequences and subdivide that large set using clustering and pattern recognition of full sequences. SCI-PHY starts with a multiple sequence alignment, builds a hierarchical tree using agglomerative clustering, and identifies the point at which to prune the tree. SCI-PHY includes phylogenetic details in the clustering. ASMC starts with a PFAM family, uses modeling and analysis of specificity determining positions (SDPs) to cluster the PFAM family, and structural modelling to create active sites; ultimately structural comparisons are performed to identify functional groups. FunFHMMer starts with and clusters a CATH-Gene3d superfamily. Essentially, FunFHMMer builds weighted HMMs of the identified clusters, so new members of each group can be identified. Both ASMC and FunFHMMer identify SDPs or mechanistic determinants that are weighted heavily in creating profiles. Remaining challenges focus on determining when subdivision is complete and identifying the SDPs more automatically.

The method described here, MISST (Multi-level Iterative Sequence Searching Technique), presents a novel approach to identifying functionally relevant clusters. Previous methods start with the complete superfamily and divisively cluster that superfamily, while the current method begins with a few examples and agglomeratively builds the isofunctional clusters from those representatives. To define groups in MISST, we build on the observation that suggests if a group is isofunctional, a DASP search using that group as the input profile self-identifies its members and no other proteins, while groups that are not isofunctional do not self-identify in this way [52]. That is, a group of proteins is deemed a functionally relevant cluster if a database search (using DASP) returns all proteins in the group at significant scores and no (or few) other proteins at significant scores (within a range of uncertainty). The iterative searches of MISST are built on this observation.

The first step in this approach is to identify the starting set of isofunctional clusters, a process called TuLIP (Two-Level Iterative clustering Procedure), during which proteins of known structure that share common active site features are clustered [52]. This process is also built on the same premise: an isofunctional cluster is one that self-identifies in a DASP search. Briefly, TuLIP starts with all structures from a protein superfamily and iteratively subdivides those into smaller and smaller groups based on active site features. At each iteration, each cluster is used in a DASP search of the sequences in the PDB. For each cluster, if the DASP search self-identifies–that is all proteins in the cluster are identified in the search and nothing else–that cluster is deemed a functionally relevant group. All clusters that do not pass this criterion are further subdivided and searched again. Results on the enolase superfamily demonstrate that TuLIP does identify the functionally relevant subgroups and families [52].

In this work, a comprehensive atlas of the Prx superfamily is identified through application of the TuLIP and MISST processes. Four functionally relevant clusters were identified by TuLIP from the known Prx structures. Through MISST iterations, sequences are added to the groups and the four clusters are subdivided into six clusters which correspond to the six expertly identified functionally relevant groups, even though this expert information of six groups was not input into the process. Because TuLIP and MISST involve iterative DASP searches, a modified process, DASP2, was used in this work. DASP2 database search results are essentially identical to DASP search results, however DASP2 is significantly more efficient than DASP [53].

This agglomerative and divisive approach allowed us to assign molecular functional detail to over 38,000 sequences, many of which were previously uncharacterized or annotated as a general Prx (or one of its synonyms). The current work suggests the feasibility of automation of MISST. Though more testing and validation is required, the MISST process should be generally adaptable for the analysis of other protein superfamilies to produce high-quality molecular function annotation and identification of isofunctional clusters within the protein universe.

## Results and Discussion

### Using TuLIP, Prx proteins of known structure are clustered based on active site features

**Fig 2:**
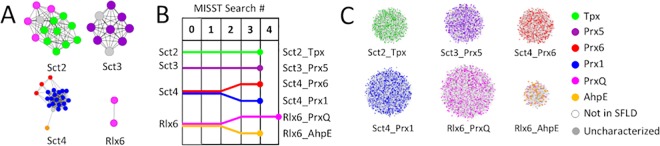
Four TuLIP groups split into six functionally relevant groups after five MISST iterations. (A) The four TuLIP groups are represented by networks in which each node represents a Prx protein of known structure. Edges are pairwise profile scores (as defined in [54]) and node colors represent expert functional annotations (see legend). (B) A dendrogram of the iterative MISST process illustrates how the initial TuLIP groups evolved into the final MISST groups. Vertical lines represent GenBank searches and dendrogram lines are colored based on the majority subgroup in each MISST cluster. Dendrogram branches represent the cluster subdivision via PSSM Analysis. The circle at each line terminus represents the iteration at which the group met self-identification criteria (see Methods). (C) The final six Prx groups are represented as networks in which nodes represent the proteins and edges represent the DASP2 search scores from the final search; the nodes are colored by expert subgroup annotation previously defined [35].

Identification of functionally relevant clusters among proteins of known structure is the first step in our process and is accomplished using TuLIP, a two-stage approach to clustering structures based on active site features [52] (see Methods for details). TuLIP identifies four functionally relevant clusters from 47 non-redundant peroxiredoxin (Prx) structures: three clusters (Sct2, Sct3, and Sct4) during the first stage and one (Rlx6) during the second stage (Fig 2A).

**Fig 3:**
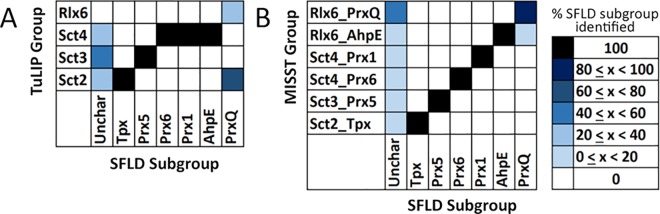
TuLIP- and MISST-identified groups correspond well with expertly-identified subgroups. TuLIP (A) and MISST (B) groups are shown on the y-axis and compared to the six known subgroups on the x-axis. Grid fill color denotes the percent of protein structures (A) or sequences (B) in each SFLD subgroup identified by each TuLIP (A) or MISST (B) group, according to the legend. The MISST heat map contains all sequences identified with a DASP2 search score ≤1e-14.

A good, though not perfect, correspondence is observed between expertly-identified subgroups, as deposited in SFLD, and TuLIP-identified groups (Fig 3A). Prx5 maps one-to-one to TuLIP group Sct3. TuLIP group Sct4 contains all proteins in three Prx subgroups: AhpE, Prx1, and Prx6, a result suggesting similar active site features, which is, indeed, observed (S1 Fig). Prx1 and Prx6 had previously been identified as being closely evolutionarily related, as well [33]. All Tpx proteins are identified in TuLIP group Sct2; Sct2 also contains four PrxQ proteins (Fig 3A). The two other PrxQ structures were grouped into their own cluster, Rlx6. This subdivision of the PrxQs of known structure was previously observed in hierarchical clustering of active site signatures [35]. Hierarchical clustering based on the canonical Prx active site motif (S1 Fig) suggests that residue differences at the PrxQ active site of proteins of known structure are driving this subdivision.

The TuLIP clustering results are not unexpected from the limited dataset of known structures and what is known about functional similarities. However, the results do present a challenge for the agglomerative and iterative process of searching sequence space: an ideal process would subdivide Sct4 into the expertly identified functionally relevant clusters and would recombine the PrxQ subgroup.

### Five iterations of the agglomerative search process, MISST, identify the six known functionally relevant Prx groups

**Fig 4:**
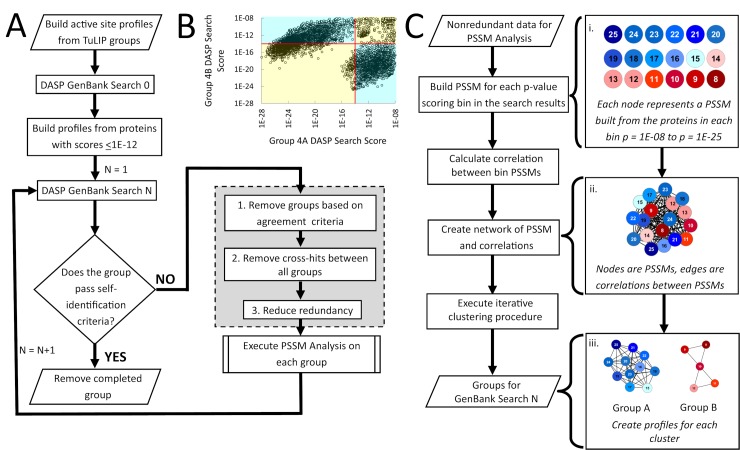
MISST and PSSM Analysis flowcharts describe the process of agglomerative identification of sequences as members of functionally relevant groups. (A) Flow chart of the MISST process for identifying functionally relevant groups within a protein superfamily. (B) An illustration of the agreement criterion: a scatterplot of all proteins identified by DASP2 searches using two ASPs, Group 4A and Group 4B, that were subdivided in the previous MISST iteration. Red lines indicate the significance threshold used to label proteins as “significant” or “not significant” in each group. Sequences in the yellow quadrants are those identified in both searches at similar (significant or not) DASP2 scores. Those sequences in the cyan quadrants differ in significance. This metric is used to determine if a group that is subdivided by PSSM Analysis produces truly distinct search results. (C) Flow chart of PSSM Analysis for identifying when and how to divide clusters into functionally relevant groups.

MISST is an iterative search process developed to be both agglomerative and divisive. That is, the process was developed to add (agglomerate) sequences containing similar functional site features to each TuLIP group and to subdivide TuLIP groups when functional site features suggest distinct clusters. As an illustration, MISST should identify the two groups represented in the ASP in Fig 1B without curator intervention. The MISST process is outlined in Fig 4 and described in detail in Methods. Briefly, the process involves iterative DASP2 searches of GenBank, each followed by evaluation for cluster division, combination, and self-identification. DASP2 is a more efficient version of the DASP sequence-searching method that focuses not on the complete protein sequence, but rather only on a protein’s functional site features [37,38,55]. Groups defined by MISST should, thus, be identified and subdivided based on their mechanistic differences. Notably, no step in the MISST process requires human evaluation—the process should be automatable, although adjustment of two parameters may be needed once the process is automated.

ASPs were created from sequences in each of the four TuLIP-identified groups: Sct2, Sct3, Sct4, and Rlx6 (profiles are provided in S1 File). Each ASP was used as input into an iterative process of DASP2 GenBank searches (see MISST flow chart, Fig 4A). Following each iteration, each group was evaluated for self-identification (Fig 4A) and need for subdivision (Fig 4C). If a group self-identifies, it is removed from the iterative process and set aside for final analysis. For all other groups, a new ASP is created from functional site pseudo-signatures (see Methods) of sequences identified at scores ≤1e-12 (Search0) or ≤1e-14 (subsequent search iterations; see Supplemental Methods in S3 File for justification, validation, and broader applicability of these score thresholds).

Five search iterations (Search0 through Search4) were performed (Fig 2B). All groups satisfied self-identification criteria after Search3 except Rlx6_PrxQ which satisfied the criteria after Search4. Through the iterations, sequences were added to each group and the four original TuLIP groups were divided into six. The process of adding sequences and splitting groups is represented in the dendrogram in Fig 2B; proteins found in the final groups are visually represented as networks in Fig 2C. Qualitatively, the six groups correspond almost perfectly with the six functionally relevant groups previously identified by experts [35] (Fig 3B).

These searches identified 38,739 sequences (Table 1) in six groups (DASP2 score threshold ≤1e-14). Proteins identified in each cluster are provided in S2 File. 6,855 of these proteins are annotated in SFLD to the subgroup matching the MISST group [41]. 30,096 proteins were not previously identified by a single DASP search [35] or by SFLD HMM analysis (Table 1); new sequences were identified due in part to their absence from the GenBank database during earlier analyses and to the more robust analysis method used here. To ascertain whether all 38,739 proteins are likely Prx superfamily members, we determined how many contained the canonical Prx active site motif **P**xxx(**T/S)**xx**C**_**P**_ [3,56,57]. Across all searches, this fragment is found in 99.3% of all MISST-identified sequences, indicating almost all sequences likely belong to this superfamily.

**Table 1:** MISST-identified group members and mapping to SFLD subgroups.

MISST Group	SFLD Subgroup Mapping	Total Number of Unique sequences ≤1e-14^1^	Identified Proteins in Mapped SFLD Subgroup^2^	Percent Subgroup Coverage^3^	Number of SFLD Uncharacterized sequences^4^	Number of sequences not in SFLD^5^
Sct2_Tpx	Tpx	4930	860	90.1	244	3826
Sct3_Prx5	Prx5	5434	1039	97.8	252	4143
Sct4_Prx6	Prx6	5212	942	96.6	127	4143
Sct4_Prx1	Prx1	9660	2130	95.7	289	7241
Rlx6_PrxQ	PrxQ	12,014	1786	92.1	739	9489
Rlx6_AhpE	AhpE	1489	98	87.5	83	1254
**TOTAL**		38,739	6855	94.3	1734	30,096

^1^Number of GIs identified ≤1e-14 in each MISST group after cross-hit analysis (Fig 4A) has been completed.

^2^Number of GIs identified ≤1e-14 in each MISST group that are annotated in SFLD to the mapped subgroup.

^3^Percent of the SFLD subgroup identified by MISST.

^4^Number of GIs identified ≤1e-14 in each MISST group annotated to the Prx superfamily by SFLD but not assigned to a subgroup.

^5^Number of GIs identified ≤1e-14 in each MISST group not in SFLD.

We next explore how the MISST process agglomerates sequences and subdivides groups. We then quantitatively compare the MISST-identified groups to the previously identified sequences. Because MISST utilizes DASP2 with its focus on functional site features as the search mechanism, we can hypothesize mechanistic determinants important for each group’s function and compare the functional site features of these expanded groups to those described by experts [35,58].

### MISST agglomerates functionally related sequences to produce the Sct3_Prx5 subgroup

MISST iterations initiated with seven Prx5 proteins in TuLIP group Sct3 ultimately identify 5434 proteins. This coherent group was not further split by PSSM analysis (Fig 2B, purple dendrogram branch), likely because of the strong intragroup active site similarity. 1039 of the MISST-identified proteins are identified as Prx5 sequences in SFLD, representing 97.8% coverage (recall). The Prx5 proteins deposited in SFLD were identified through one DASP iteration [35]; a few more were added through the SFLD curation processes. This group contains no proteins from any other Prx subgroup (Fig 3B); consequently, Sct3 is mapped to Prx5 for subsequent analysis and herein called Sct3_Prx5.

**Fig 5:**
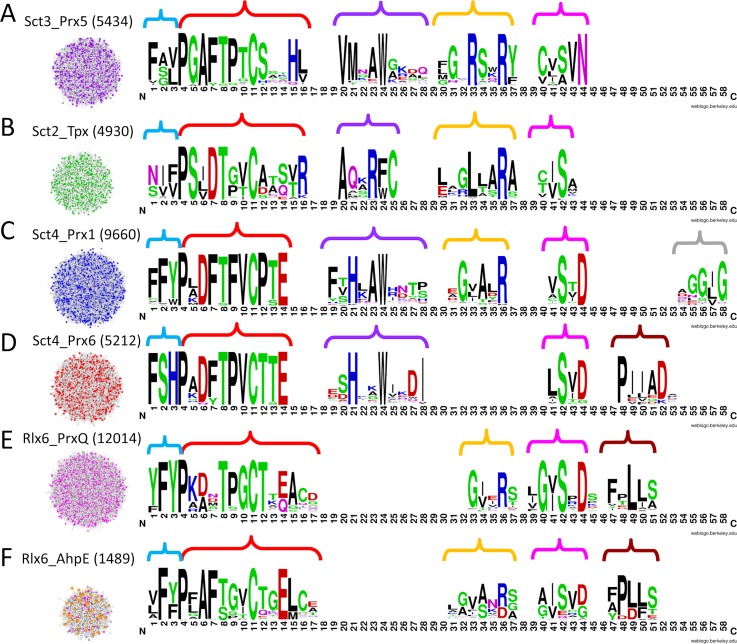
Signature conservation graphs highlight potential specificity determining positions (SDPs) in each of the six Prx subgroups. Pseudo-signatures (see Methods) for the significantly scoring proteins (post cross hit analysis) in each MISST group were used to construct signature conservation graphs (signature logos of the active site profiles). Letter height indicates the residue conservation in that position. Colored braces indicate motifs discussed in the text. The clusters on the left show the proteins used to create the signature logos, colored by previously defined subgroup; the number in parenthesis represents the number of proteins in each cluster. The signature logos were created using WebLogo version 2.8.2 with default settings and with the y-axis not shown.

Sct3_Prx5 includes 252 proteins identified in SFLD as belonging to the Prx superfamily, but uncharacterized with respect to subgroup; thus, the functional subgroup of these proteins can now be defined more precisely. 4143 Sct3_Prx5 proteins were not previously identified as Prx5 (Table 1) demonstrating that, if the new identifications are correct, search iterations of MISST add significantly to our knowledge of functionally related proteins. Consequently, the probability that these proteins are actual Prx5 proteins was evaluated by determining the presence or absence of the Prx5-specific active site motif **P**(G/A)A(F/Y)**(T/S)**(P/G)x**C**_**P**_ [9] (Fig 5A, part of red brace). 97.4% of all Sct3_Prx5 sequences contain this motif. The percentages do not differ between previously known and newly identified proteins: 98.2% of previously identified Prx5 proteins, 96.4% of Prx sequences in SFLD that are uncharacterized relative to subgroup, and 97.2% of new (non-SFLD) proteins contain the motif. Given that the percent of both knowns and new proteins containing this motif is similar, there is high probability that the MISST iterations are consistently identifying proteins that belong to the Prx5 functional family.

To quantitatively evaluate sequence identification, F-measure, the harmonic mean of precision and recall [59], was calculated for Sct3_Prx5 sequences. For this analysis (and similar analyses of other groups), Prx proteins in SFLD are the known sequences; “positive” sequences are the proteins in the subgroup under consideration, while “negative” sequences are Prx sequences in all other subgroups. Thus, if a known Prx5 was identified by MISST, a true positive was counted. If a sequence from another Prx subgroup was identified as part of Sct_Prx5, a false positive was counted. False negatives were Prx5 sequences identified in the previous work [35], but not identified in this search. A true negative is counted if MISST did not identify Prx sequences known to be members of other Prx subgroups. Sequences identified by MISST, but not by previous methods, were not included in this analysis, as their assignment as true or false positives or negatives could not be evaluated. This is a difference between MISST and other methods: instead of subdividing a superfamily in which all proteins are thought to be known at the start [49–51], MISST agglomeratively adds proteins from the database and subdivides the groups.

**Fig 6:**
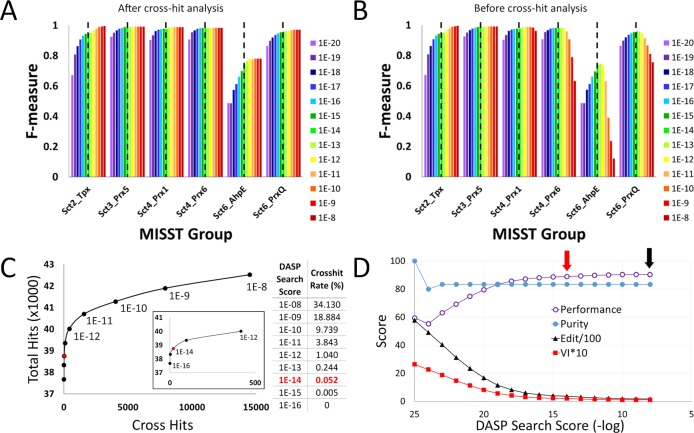
Quantitative analysis shows final MISST groups are distinct and correspond well with previously identified proteins. F-measure, the harmonic mean of precision and recall, is calculated for each of the six MISST groups at each DASP2 search score threshold (A, B). DASP2 score thresholds are represented by different colored bars, according to the legend, from most significant (purple) to least significant (red); dashed black lines indicate the significance threshold ≤1e-14. F-measure was calculated both before executing cross hit analysis (B) and after executing cross hit analysis (A) (see Methods). The number of cross hits, or GIs identified by more than one MISST group, is plotted against the number of unique GIs identified by all six MISST groups at each DASP2 search score threshold (C). The inset is a magnified view, showing only thresholds ≤1e-12 to ≤1e-16. A table shows the cross hit rate as a percentage, which is the number of cross hits divided by the number of total unique hits, at each score threshold. In both the graph and the table, the significance threshold ≤1e-14 is highlighted in red. The graphs and table in both (B) and (C) were constructed prior to completing cross hit analysis (see Methods). Performance, edit distance, VI distance and purity values (details in S3 File) are shown for each DASP2 search score threshold from ≤1e-8 to ≤1e-25 (D). These scores were calculated after executing cross hit analysis. The black arrow highlights peak performance and the red arrow highlights the significance threshold ≤1e-14.

F-measure analysis demonstrates the high quality of assignments to Sct3_Prx5 (Fig 6A): the F-measure is 0.99 at the DASP2 search score threshold (≤1e-14, dashed line Fig 6A). As the DASP2 score threshold becomes more significant, recall gradually decreases (as some proteins are missed); however, precision never drops below 1 for Sct3_Prx5. Neither precision nor recall decrease in this group as the DASP2 score threshold becomes less significant (yellow, orange, and red bars, Fig 6A), indicating no false positives are identified at ≤1e-8, even prior to cross hit analysis (Fig 6B).

Detailed analysis of the Sct3_Prx5 functional site pseudo-signatures identify mechanistic determinants distinctive to this subgroup (Fig 5A; structures in S2 Fig). These determinants were not identified *a priori* as input. The Prx active site motif includes elements distinctive to the Sct3_Prx5 subgroup: **P**(G/A)A(F/Y)**(T/S)**(P/G)x**C**_**P**_ (bold indicates residues almost invariant across the superfamily; [9,35]). Outside of this motif, two defining features are observed: His is almost invariant at signature position 15 (Fig 5A red brace) and a pair of Arg residues (RSxR(Y/F)) at positions 33–37 (yellow brace). The second of these conserved Arg residues is the one recognized to play a major role in activating the peroxide substrate for–O-O–bond scission at the Prx active site [9]. In the structure 1TP9, the side chain of the His residue conserved in Prx5 proteins is hydrogen bonded to the side chain of this invariant Arg (signature position 36; S2A Fig). The location of these side chains in the active site near the C_P_ suggests a role in mechanism, perhaps with the His playing a role in proton transfer. Reasonably well conserved motifs in the pseudo-signatures of this subgroup also include VMxxW at signature positions 20–24 and (C/V)(V/L/I)(S/A)VN at signature positions 39–43 (Fig 5A, purple and fuchsia braces, respectively). The Cys in this second fragment is found in 76% of sequences; 19% of sequences have Val at this position. Further, phylogenetic evidence suggests conservation of this Cys, which sometimes serves as the C_R_, may be based on phylogeny (S3 File; S3 Fig, red brace).

Starting with just nine structures, MISST agglomerates sequences into a coherent Prx5 cluster. Even though PSSM analysis was performed at each iteration, the Sct3_Prx5 group did not split, suggesting that the PSSM approach does not split functionally relevant clusters.

### MISST eliminates sequences that are less functionally related to create a coherent Sct2_Tpx subgroup

**Fig 7:**
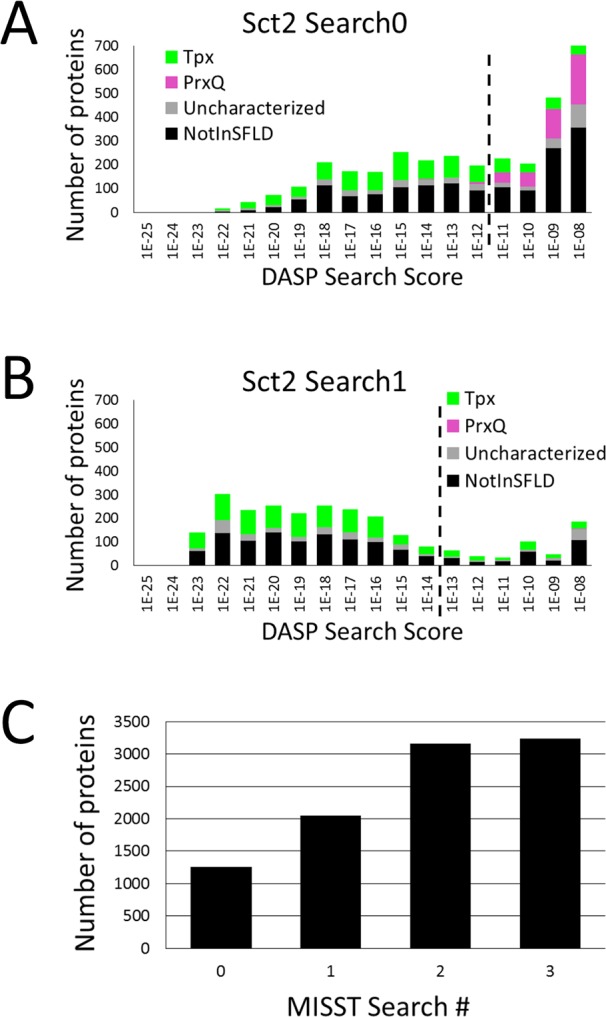
Agglomeration of Tpx sequences and loss of PrxQ sequences in Sct2_Tpx during MISST search iterations. The proteins identified in Sct2_Tpx Search0 (A) and Search1 (B) are displayed as histograms with bars colored to show previously known functional groups. Dotted black lines signify the DASP search score threshold of ≤1e-12 for Search0 and ≤1e-14 for Search1. (C) The number of total proteins identified by Sct2_Tpx at significant DASP2 search scores is shown for searches 0 through 3.

Sct2 was originally comprised of four PrxQ sequences and nine Tpx sequences (Fig 3A). Known Tpx structures contain the resolving cysteine, C_R_, in the α3 helix. The C_R_ is not found in a consistent location in the four TuLIP-identified PrxQ proteins. Using the Sct2 TuLIP group as MISST input illustrates sequence agglomeration and increasing coherence within a cluster, despite the group’s initial heterogeneity. At Search0, known PrxQ proteins are identified at less significant DASP2 search scores than the Tpx proteins (Fig 7A). By the second iteration (Search1) known PrxQ proteins are not identified (Fig 7B). Iterative DASP2 searches produce more robust profiles and each successive search produces a more coherent set of sequences that exhibit common active site features.

With each iteration, additional Tpx proteins accumulate, with a plateau reached in Search2 and Search3 (Fig 7C). At Search3, ≥ 95% of sequences used as input to Search3 and ≤ 15% new sequences were identified at significant DASP2 scores (≤1e-14) in the GenBank Search3; thus, self-identification criteria were satisfied following Search3 (Fig 2B, green dendrogram branch). At this point, the group was homogeneous for Tpx proteins and is thus called Sct2_Tpx.

The final Sct2_Tpx cluster contains 4930 sequences—860 are in SFLD and annotated to the Tpx subgroup, 244 are marked as Prx-uncharacterized in SFLD, and 3826 are not in SFLD (Table 1). F-measure shows high precision and recall values for Tpx proteins (Fig 6A). After this group satisfied the self-identification criteria, no false positives were identified even at less significant scores of ≤1e-8. 860 SFLD Tpx sequences represent 90.1% coverage (recall) of known subgroup members; F-measure is 0.95 at the DASP2 score threshold of ≤1e-14 (Fig 6A).

Of the final sequences in this Sct2_Tpx group, 98.5% contained the Prx active site motif distinctive for this subgroup: **P**S(I/L/V)D**T**x(V/T/I)**C**_**P**_ (Fig 5B, red brace), which refines the motif determined from the previously identified smaller dataset [9,35]. The sequences are 99.94% bacterial (S4A Fig), consistent with what was previously reported on the smaller dataset [58].

Additional mechanistic determinants can be hypothesized for the Sct2_Tpx subgroup. Signature positions 15 and 16 are distinctive in this group: a branched residue (Val or Thr) followed by Arg or Lys (Fig 5B, red brace). A distinctive AxxR(F/W)C motif is observed at signature positions 20–25 (Fig 5B, purple brace). This conserved Cys is the C_R_ in helix α-3. As in Sct3_Prx5 and Sct4_Prx1, the nearly invariant (99.3%) Arg at signature position 36 is the active site residue required for efficient catalysis [9,60]. In the structure 3P7X, the side chain of this Arg is hydrogen bonded to C_P_. It is preceded by a very well-conserved Leu at signature position 33, the only subgroup with a well conserved hydrophobic residue at this position (Fig 5B, yellow brace). Both Arg (gray) and Leu (black) extend towards C_P_ (S2B Fig). Finally, the Sct2_Tpx subgroup contains a Ser that is almost invariant at signature position 42 (Fig 5B, fuchsia brace). These residues are proximal to the active site, suggesting a functional role (S2B Fig).

This example illustrates how the iterative MISST process creates more coherent groups, even when the original TuLIP group is composed of two subgroups. While the PrxQ structures were not present in the final Sct2_Tpx MISST group, this subgroup was not lost in the MISST process. As discussed subsequently, the PrxQs were identified as a subdivision of the Rlx6 group.

### PSSM Analysis subdivides Sct4 into Prx1 and Prx6 subgroups

The clustering process described herein starts with proteins of known structure; however, the structure database is a very limited representation of the sequence space universe. Because of this limitation, TuLIP sometimes combines multiple subgroups into one cluster [52], as is the case with Sct4, which contains both Prx1 and Prx6 proteins. Consequently, any agglomerative process aimed at identifying functionally relevant groups must recognize the need for cluster subdivision. PSSM Analysis was developed as an automatable process to do just this.

PSSM Analysis is performed at each MISST iteration after the first (Fig 4A) using the outlined process (Fig 4C; details in Methods). Essentially, the active site pseudo-signatures identified in the GenBank search are used to quantitatively determine if and how the group should be subdivided. If subdivision is required, two new ASPs are created from the appropriate pseudo-signatures. These ASPs are input to a DASP2 search of GenBank. Search outputs are compared to verify the groups are, in fact, unique. Notably, PSSM Analysis was performed at each search iteration for both the Sct2 and Sct3 MISST groups, but distinct, functionally relevant groups were not identified within either group.

The TuLIP-identified Sct4 group includes all known structures from the Prx1, Prx6, and AhpE subgroups (Figs 3A and 4A). At Search1, PSSM Analysis identifies two groups; these groups evolve distinctly through subsequent search iterations (Fig 2B, red and blue dendrogram branches). Notably, though the AhpE subgroup is not identified in Sct4 after Search1, the AhpE subgroup is not lost. It is ultimately identified in Rlx6 using this same PSSM Analysis procedure (discussed subsequently).

**Fig 8:**
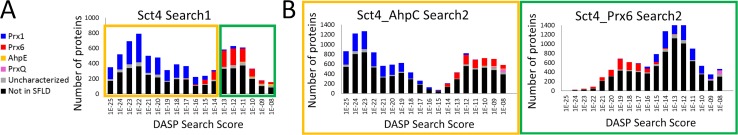
PSSM Analysis subdivides Sct4 into Prx1 and Prx6 groups based on distinctive active site features. (A) A score distribution of the Sct4 Search1 results is shown with bars colored based on known functional annotation. The yellow and green boxes identify the groups distinguished by PSSM Analysis. (B) Search2 score distributions show the results of the subsequent MISST iteration, in which profiles of sequences in each of the yellow and green boxes were created and used in separate searches.

Analysis of each search iteration provides insight into the PSSM Analysis of Sct4 (Fig 8). The Search1 DASP2 score distribution is bimodal—Prx1 sequences at more significant and Prx6 sequences at less significant DASP2 search scores (Fig 8A, blue and red bars). PSSM Analysis correctly identifies these two groups (Fig 8A, yellow and green boxes). One ASP is created each for sequences in the yellow and green boxes; each ASP is used in Search2 of GenBank. Prx1 and Prx6 sequences are identified distinctly in Search2 (Fig 8B, Search2 distributions). After just one more GenBank search iteration (Search3), each group passes self-identification criteria.

Ultimately, 9660 and 5212 sequences are identified at significant DASP2 scores in Sct4_Prx1 and Sct4_Prx6, respectively (Table 1). Of the proteins annotated in SFLD, 96.6% of Prx6 proteins and 95.7% of Prx1 proteins are identified (Table 1). Both searches identify Prx sequences annotated as Prx-uncharacterized in SFLD: 127 and 289 are identified as part of Sct4_Prx6 and Sct4_Prx1, respectively. Finally, 4143 and 7241 GenBank sequences not annotated in SFLD were identified as Sct4_Prx6 and Sct4_Prx1 members, respectively (Table 1).

The Prx active site motifs for Sct4_Prx1 and Sct4_Prx6 are distinct: **P**xDF**(T/S)**FV**C**_**P**_ and **P**x(D/N)(F/Y)**T**PV**C**_**P**_, respectively (Fig 5C and 5D, red braces). 93.8% and 96.7% of all sequences in Prx1 and Prx6, respectively, exhibit these motifs, demonstrating that MISST iterations and the PSSM Analysis distinguish these small active site differences. F-measure at the score threshold of ≤1e-14 is high for both groups: 0.98 at a DASP2 score threshold of ≤1e-14 for each (Fig 6A). Thus, PSSM Analysis can effectively subdivide one group into two functionally relevant clusters.

As with the other groups, we can identify mechanistic determinants that distinguish Sct4_Prx6 and Sct4_Prx1. A key distinguishing feature is the TFVC versus TPVC for Prx1 and Prx6, respectively: this one residue in the canonical Prx active site motif distinguishes these two subgroups (Fig 5C and 5D, red brace; S2 Fig, cyan side chains). Another distinguishing feature is a Phe-Tyr (Prx1) compared to Ser-His (Prx6) at signature positions 2 and 3 (Fig 5C and 5D, blue brace). In 2V2G, this His is in the active site, near the C_P_ (S2D Fig, yellow side chains). Again, Arg at position 36 in Prx1 is the active site residue required for efficient catalysis; the fragment containing this Arg is not part of the Prx6 signature. In both subgroups, the almost invariant Ser (at signature position 42) and the almost invariant His (at signature position 21) form a potential path for proton transfer in these subgroups (S2C and S2D Fig, light pink side chains). C_R_ is not observed within the Prx1 group profile because it is contributed from a different chain (the partner subunit of the dimer). There is no C_R_ in most Prx6 members [35]. Interesting phylogenetic observations at specific positions, including the well-known GG(L/I/V)G motif [31], are discussed in S3 File.

Previous sequence analysis methods identified Prx1 and Prx6 as only one group, which the authors named Prx4 [33]. Subsequent expert analysis clearly showed that Prx6 was a distinct functionally relevant group [35]. MISST, a method that focuses on differentiating active site features, has accomplished that which was previously accomplished only by expert curation—to divide these two closely related isofunctional clusters without human curation. This opens the exciting possibility of functionally relevant clustering of superfamilies for which functional groups are not known.

### PSSM Analysis subdivides Rlx6 into PrxQ and AhpE subgroups

PrxQ and AhpE were members of original TuLIP groups, but were lost from Sct2 and Sct4 searches, respectively, during MISST iterations. TuLIP group Rlx6 contained two of the six PrxQ structures known at the time this research was completed. The task is even more difficult because AhpE is a very small subgroup containing only 25 proteins in 2011 [35] and 112 in the current SFLD; previously, these proteins were found in only one class of bacteria (actinobacteria) [58]. Only one structure is available in the PDB database. Are these groups that are less well represented by structures identified through the iterative MISST process applied to TuLIP group Rlx6? The answer to this important question is yes.

Analysis of the Rlx6 MISST search iterations illuminates how AhpE and PrxQ sequences are identified and subdivided (Fig 2B, pink and yellow dendrogram branches). The Search0 ASP input contained only two PrxQ proteins (Fig 2A); Search0 output contained mostly PrxQ proteins, with a few AhpE proteins (not shown). Per the MISST process (Fig 4), an ASP was created for sequences identified at a DASP2 score threshold of ≤1e-12. This ASP was input to Search1.

**Fig 9:**
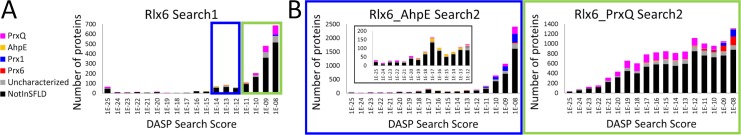
PSSM Analysis subdivides Rlx6 into AhpE and PrxQ groups. (A) The DASP2 score distribution of the Rlx6 Search1 results is shown with bars colored by known functional annotations (see legend). The blue and green boxes represent the two groups identified by PSSM Analysis. (B) The DASP2 score distributions that result from Search2, which uses as input the ASPs composed of the proteins in the blue and green boxes from (A). Search2 results illustrate the separation of the AhpE (orange) and PrxQ (pink) subgroups. An inset shows more detail for scoring bins 1e-25 to 1e-12 for the Rlx6_AhpE Search2 histogram.

Search1 output includes a small number of AhpE and PrxQ proteins at more significant scores; most PrxQ proteins are identified at less significant DASP2 scores (Fig 9A). PSSM Analysis divides Search1 sequences into two groups: AhpE and PrxQ (Fig 9A, blue and green boxes). An ASP is created for each group, and each ASP is input to DASP2 Search2. Results are distinct: one search is populated with mostly AhpE and a few PrxQ proteins, the other populated almost solely with PrxQ proteins (Fig 9B). The Rlx6_AhpE and Rlx6_PrxQ groups subsequently remain distinct (as determined by the agreement criterion; Fig 4B) and pass self-identification criteria at Search3 and Search4, respectively (Fig 2B, yellow and pink dendrogram branches).

The two groups map easily to subgroups identified by experts. One, Rlx6_PrxQ, contains 12,014 sequences; 1786 of these sequences are found in SFLD, which represents 92.1% of known PrxQ proteins (Table 1). 739 sequences are annotated in SFLD to the Prx superfamily but not a specific subgroup. MISST identifies 9489 sequences in this cluster that were not previously assigned to the Prx superfamily (Table 1). Consistent with the other MISST-identified groups thus far discussed, F-measure (and, thus, precision and recall) is quite high, 0.96, for Rlx6_PrxQ (Fig 6A).

Rlx6_AhpE is by far the smallest subgroup identified by MISST: only 1489 sequences are identified in this cluster. 98 of those proteins are currently annotated as AhpE in SFLD, which represents 87.5% of the 112 known AhpE proteins. 1254 Rlx6_AhpE proteins were not previously identified as Prxs (Table 1). Notably, F-measure for this cluster is not as strong as the other MISST-identified groups—only 0.74 at the DASP2 score threshold of ≤1e-14. In addition, the F-measure is never above 0.78, even at less significant score thresholds (Fig 6A). Detailed analysis explains this result. There are 112 nonredundant AhpE sequences in SFLD. At thresholds of ≤1e-14, ≤1e-12, and ≤1e-10, we identify 98, 107, and 108 of them, respectively; thus, recall is high, at 87.5% at ≤1e-14 and increases to 96.4% at ≤1e-10. However, 54 Rlx6_AhpE proteins identified at the DASP2 search score threshold of ≤1e-14 were previously identified as PrxQ subgroup members [35]. These proteins decrease the precision of the result. The question of functional assignment of these 54 sequences is an important one; these sequences are listed in S2 File and discussed subsequently.

The two clusters derived from Rlx6 exhibit common active site features, such as the Phe at signature position 2 (Fig 5E and 5F, blue brace), which is highly conserved in both groups. However, the Prx active site motif is distinct between Rlx6_BCP and Rlx6_AhpE, including the canonical Prx active site motif: **P**(K/A/R)(D/A)x**T**xG**C** and **P**xAF**(T/S)**xx**C** for Rlx6_PrxQ and Rlx6_AhpE, respectively (Fig 5E and 5F, red braces). 90.2% of proteins identified in Rlx6_PrxQ contained its motif, while 94.2% of the Rlx6_AhpE sequences, including 92.9% of those previously identified as AhpE and 88.9% of those previously annotated as PrxQs, contain its motif. Notably, Rlx6_PrxQ is the only subgroup with a Gly strongly conserved immediately preceding C_P_, which might suggest unique conformational or dynamical motion associated with PrxQ function.

Other positions also distinguish these two Rlx6-derived groups. Glu is invariant at signature position 14 in Rlx6_AhpE, while the residue can be either Glu or Gln in Rlx6_PrxQ. The final two active site fragments are also distinct (Fig 5E and 5F, fuchsia and purple braces, respectively). A G(V/I)SxD motif at positions 40–44 and a Leu at position 49 are strongly conserved in Rlx6_PrxQ. Notably, the invariant Gly, Ser, and Asp of the G(V/I)SxD motif are all in the 5ENU active site, along with the conserved Leu. These distinctive features suggest that, indeed, these two subgroups are functionally distinct.

**Fig 10:**
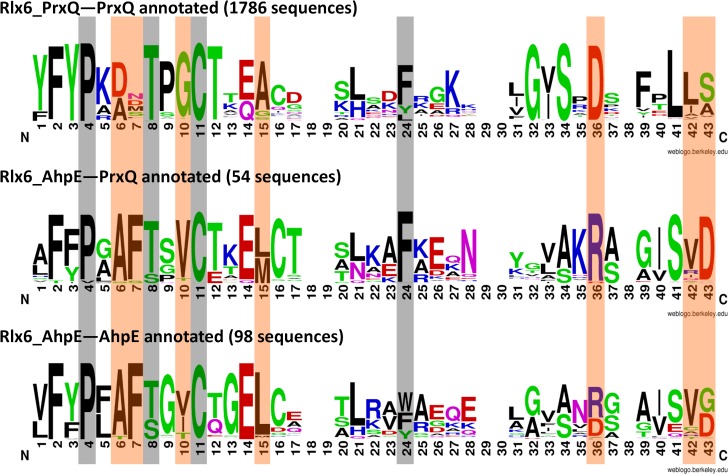
Comparison of AhpE and PrxQ signatures suggests why 54 previously annotated PrxQ proteins are identified in the Rlx6_AhpE MISST group. Signature conservation graphs were made for all proteins previously annotated as PrxQ in the Rlx6_PrxQ group and all proteins previously annotated as PrxQ or AhpE in the Rlx6_AhpE MISST group. Gray highlights represent the key residues used to initiate TuLIP. Orange highlights represent positions in which Rlx6_AhpE proteins annotated as PrxQ share more similarity with the AhpE subgroup than the PrxQ subgroup. Signature conservation graphs were made using Weblogo version 2.8.2 [61] with default settings, including small sample correction.

The question remains: what is the correct functional classification of the 54 sequences previously classified as BCP [35] and classified by MISST as AhpE? A closer analysis of the active site signatures may explain the unexpected clustering. Signature logos were created for the 1786 Rlx6_PrxQ sequences that were previously annotated as PrxQ, the 98 Rlx6_AhpE proteins previously annotated as AhpE, and the 54 Rlx6_AhpE sequences previously annotated as PrxQ (Fig 10). Multiple positions in the active site signature illustrate why the 54 sequences previously annotated PrxQ are now identified in the AhpE MISST group, including a strongly conserved Ala-Phe dyad in the canonical PrxQ active site motif (**P**xAF**(T/S)**xx**C**), a conserved Val or Ile immediately preceding C_P_, and four other positions (Fig 10, orange highlights). These results demonstrate the DASP2 method identified these 54 proteins in the Rlx6_AhpE subgroup because of common features at the active site. Further, specific residues can be identified that distinguish bacterial (81%) and archaeal (19%) proteins in the Rlx6_AhpE subgroup (full discussion in S3 File). The biological relevance of these observations remains to be determined.

In conclusion, the original Rlx6 TuLIP group contained just two of six PrxQ structures, and the lone AhpE structure was in Sct4, not Rlx6. Despite only one known AhpE structure, PSSM Analysis and iterative DASp2 searches extracted the AhpE functional group from the Rlx6 search results. These results demonstrate the MISST process can identify functional groups for which structural representation is limited. This is an important result, as many protein superfamilies do not contain comprehensive structural representation in all functional families. Over all six Prx functional groups, the iterative MISST process meets the challenges presented by the TuLIP results: all six Prx subgroups were identified in a robust and comprehensive fashion, even though not all groups were well-represented in the structure database.

### Quantitative analyses of sequences in six MISST-identified groups show high quality of MISST performance

Results presented thus far demonstrate MISST can both add sequences to functionally relevant groups and subdivide groups into clusters exhibiting distinctive functional features. F-measure (precision and recall, Fig 6A) was described for each group individually. Further quantitative comparison between groups, including cross-hit counts and measures of performance, are essential to determine if these groups are distinct and functionally relevant.

Cross-hits are defined as the same sequence identified in more than one MISST group at a given DASP2 search score threshold. This analysis demonstrates discreteness of MISST groups. In creating the final groups, a cross-hit analysis similar to that previously described [35] is performed (see Methods). Only 20 proteins are removed in this final cross-hit analysis; the identities of the proteins which cross-hit are listed in S1 Table.

To understand the discreteness of the MISST-identified groups, the correlation of cross-hits (counted prior to this final cross-hit analysis) with the DASP2 search score threshold was evaluated (Fig 6C). At DASP2 search score thresholds of ≤1e-16 and more significant, all groups are distinct—the number of cross-hits is zero. At the significance threshold of ≤1e-14, the threshold identified as a “trusted” threshold in the work described here (see Supplemental Methods in S3 File), 20 cross-hits are identified corresponding to a cross hit rate of 0.052%, an extremely low false positive rate (Fig 6C, table and red data point).

Cross-hits increase drastically as the DASP2 search score threshold decreases in significance (Fig 6C). We can observe the evolution of the cross-hits and, thus, better understand the relationship between group active sites by analyzing “fireworks plots,” a form of network analysis (S5 Fig). At a DASP2 search score threshold of ≤1e-8, Prx5 and Tpx subgroups are most distinct and only exhibit a few cross-hits to other groups, which are mostly gone at a threshold of ≤1e-10 (S5A and S5B Fig). At a score threshold of ≤1e-12, the other four groups become more distinct (S5C Fig). At a score threshold of ≤1e-14, only twenty cross hits remain. Ten of these twenty cross-hits at ≤1e-14 are between AhpE and PrxQ (S5D Fig), indicating the functional sites of these groups are more closely related to each other than they are to the other groups, as discussed above. The other ten cross-hits are distributed between Prx1, Prx6, and PrxQ (S5D Fig).

The remaining analysis, F-measure and Performance, assumes that the expert annotations deposited in SFLD [35] are correct. These sequences were identified by a single DASP search of GenBank using expertly-created ASPs. Subsequently, sequences were added using the SFLD HMM approach. The resulting sequences were curated by hand and deposited in SFLD; these annotations are the best known molecular functional annotations for the Prx superfamily. Only 412 sequences previously identified as Prx are not identified as part of the proper MISST group (out of 7267 Prxs in SFLD with subgroup annotations). These sequences are evenly spread over the six groups and are counted as false negatives in the recall calculation of F-measure. 54 of these are the sequences previously annotated as PrxQ, but identified in this analysis as AhpE. 194 sequences were identified above the DASP2 score threshold of 1e-14. About 25 of the sequences are no longer in GenBank.

Since the 2011 analysis of the Prx superfamily, GenBank has grown from 11.9M proteins to over 54.8M proteins at the end of 2015. With this growth comes many new sequences identified in our MISST searches that are not annotated in SFLD. To quantify the performance of MISST, all sequences not annotated in SFLD were not used for the F-measure and Performance analyses as the correct annotation is unknown. (In the previous sections, we demonstrated the likelihood that these newly identified sequences were Prx by evaluating the presence and absence of the canonical Prx active site motif, **P**xxx(**T/S**)xx**C**_**P**_, as well as the active site motif associated with each subgroup.)

To analyze the overall accuracy of the MISST process, a performance score was calculated [49,50] taking into account purity, edit distance, and VI distance [62] (Fig 6D). These measures were calculated by defining the proteins in each group as TP, TN, FP, or FN; these definitions were based on the previous Prx annotation [35] (see Supplemental Methods in S3 File). Purity provides a measure of the proportion of groups which contain only one subgroup. As Rlx6_AhpE is the only group containing false positives, purity remains at 83.3% (5 out of 6 groups are pure) until highly significant DASP2 search score thresholds (Fig 6D, blue). Edit and VI distances measure how many changes are required to transform one grouping method (MISST) to another (SFLD). The high correlation between the six SFLD subgroups and the six MISST groups leads to low edit and VI distances, particularly at less significant score thresholds (Fig 6D, black and red). The increase in edit and VI distance values at more significant scores is due to the presence of “singlets,” which in this case are Prx proteins in the SFLD not identified as a member of any MISST group. Typically, edit and VI distances are used to compare two clustering methods which both start with the same set of proteins. However, MISST is an agglomerative method and does not begin with the full set of proteins; therefore, some proteins in the SFLD are not identified by MISST. Thus, as the DASP search score threshold becomes more significant, more proteins are classified as “singlets” because they are not identified in any MISST groups at the given threshold.

Purity, edit, and VI distance were combined into an overall performance measure (Fig 6D, purple). A maximal performance score of 90.3 is found at a score threshold of ≤1e-8; the performance at the threshold of ≤1e-14 is 88.9 (Fig 6D, colored arrows). Performance does not reach 100 at any point because not all known Prx proteins are identified by the six MISST groups and some PrxQ-annotated proteins are identified in the Rlx6_AhpE group. Performance increases slightly at the less significant score thresholds, simply due to the behavior of edit and VI distance with “singlets.” The value of 88.9 at a score threshold of ≤1e-14 compares well with the performance values reported for clustering of other gold-standard SFLD superfamilies (amidohydrolase, crotonase, enolase, HAD, terpene cyclase, VOC) by SCI-PHY (performance ranged from 54.99 to 91.70, with an average of 75.36) and GeMMA with a generalized cutoff (performance ranged from 53.64 to 90.70, with an average of 80.42) [49]. It is important to note that performance scores vary widely for both SCI-PHY and GeMMA, indicating more superfamilies must be tested using MISST to complete a full-scale comparison between methods. However, this initial test using the Prx superfamily demonstrates the feasibility of the current approach.

### Network analysis highlights significant differences between active site similarity and sequence similarity in Prxs

Previous work has illustrated how different comparison measures (sequence, structure, functional site) can produce different clusters within a protein superfamily [54]. Here we explore that further, by evaluating full sequence similarity between the functionally relevant MISST clusters.

**Fig 11:**
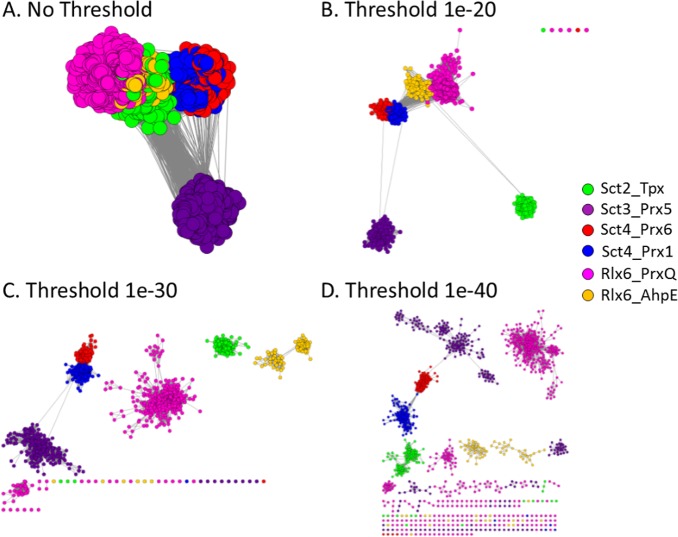
Representative network highlights sequential similarity between Sct4_Prx1 and Sct4_Prx6 MISST groups. A representative network shows all proteins identified by the six MISST groups, with one representative per 55% sequentially identical cluster. The nodes in the representative network are colored by MISST group (see legend), and the edges represent pairwise BLAST scores between the representative proteins. The network is shown with no edge value threshold (A), and e-value thresholds of 1e-20 (B), 1e-30 (C), and 1e-40 (D), where all edges with scores greater than the threshold are removed prior to applying the force-directed layout. Network visualizations were created with Cytoscape.

A representative network (RepNet) was built from the 38,739 sequences identified in the six MISST groups. Each of the 1,369 nodes represents proteins sharing 55% sequence identity; each edge represents the pairwise BLAST score (sequence comparison) between the representatives of the two nodes. Nodes are colored based on the MISST group to which the sequences belong (see Methods for more details). The network is filtered at a variety of BLAST score thresholds to visualize the full length sequence similarity among the MISST groups (Fig 11).

As the threshold for edge becomes more stringent, groups begin to separate. Notably, and as expected, the sequences within MISST groups are more similar to each other than they are to proteins from other groups. This observation is the reason that full sequence comparison methods (like BLAST) do reasonably well at identifying the superfamily level of function, like peroxiredoxin.

Notably, no single threshold can be identified to distinctly identify the six known subgroups, an illustration of why full sequence comparison methods are less successful at identifying detailed levels of molecular function, such as distinguishing between Prx1 and Prx6. At less stringent edge (BLAST score) thresholds (Fig 11B), some subgroups are indecipherable from one another (such as Prx1 to Prx6 and AhpE to PrxQ), and at more stringent thresholds (Fig 11D), some subgroups begin to split unnecessarily (such as PrxQ, AhpE, and Prx5). Unsurprisingly, the Prx1 and Prx6 subgroups are difficult to distinguish from one another until the most stringent threshold. Previous work has demonstrated that the similarity between these 2 subgroups makes it difficult to separate them based on sequence comparison alone [33]. MISST focuses on active site features to define isofunctional groups, thus eliminating reliance on full sequence comparison for detailed molecular function analysis.

### Conclusion

In this work, active site features are utilized to define functionally relevant clusters. A method, MISST, which uses self-identification of clusters to define functional relevance is introduced. The method is both agglomerative and divisive. As ASPs become more robust, DASP2 searches agglomerate more functionally related sequences. Likewise, at each stage, clusters are evaluated for the presence of groups that exhibit distinct functional site features. Functionally relevant clustering of the Prx superfamily presents several challenges for the method: How are sequences agglomerated (Tpx and Prx5)? How are clusters subdivided when they contain two distinct isofunctional groups (Prx1 and Prx6)? And, how are functionally relevant groups identified when structural representation is extremely limited (AhpE)?

A defining feature of functional annotation is the hierarchy under which groups of proteins are classified [40,41,54], and it is important to understand how the MISST results fit into a functional hierarchy. Members of the Prx functional superfamily all perform a similar redox chemistry at C_P_; differences lie in substrate recognition and details of how C_P_ is regenerated. The six expert-annotated groups of Prxs are classified as subgroups in the SFLD, which indicates that group members share more features among themselves than with members of other Prx subgroups.

MISST distinguishes these six subgroups, and members thereof, identifying the differences between the mechanisms from which hypothesis-driven experiments can be developed. As expected, many new sequences were identified—the Prx data in SFLD is from 2010 and 2016 GenBank is significantly larger. Over 99% of newly identified sequences contain the canonical Prx active site motif. Additionally, with the exception of the AhpE subgroup, the phylogenetic distribution for each subgroup is reasonably consistent with the original Prx data, as recently reported by Poole and Nelson [58].

The current work demonstrates the feasibility of this novel, agglomerative approach of using self-identification to identify isofunctional clusters. Notably, the MISST process does not require human- or expert-based analysis and is automatable, with the exception of identification of the key functional residues which are input to TuLIP. Two MISST parameters may require further adjustment to demonstrate generalizability: score thresholds and self-identification criteria. However, our work on the enolase and Prx superfamilies suggests the score thresholds are generalizable (S3 File). The feasibility of MISST is demonstrated here on the Prx superfamily. More extensive parameterization, validation, and generalizability will be demonstrated once the method is automated. Ultimately, we envision that MISST could be applied to cluster any protein superfamily automatically, thus laying the foundation for functionally relevant clustering of the universe of protein sequences.

## Materials and Methods

### Protein set—peroxiredoxins

The peroxiredoxin (Prx) superfamily contains six subgroups previously identified by expert analysis: Prx1 (formerly AhpC/Prx1), AhpE, PrxQ (formerly BCP/PrxQ), Prx5, Prx6, and Tpx [28,32,34,35]. These expertly-identified subgroups are available in PREX [39]. Curators at the Structure-Function Linkage Database (SFLD) constructed hidden Markov models (HMMs) for each subgroup and have updated the proteins in each subgroup in SFLD (Prx superfamily, EC 1.11.1.15) [40,41]. As of March 7, 2016, there were 7,267 annotated Prx proteins (unique EFDIDs) in SFLD, distributed among the subgroups as follows: 2,225 Prx1, 112 AhpE, 1,939 PrxQ, 1,062 Prx5, 975 Prx6, and 954 Tpx. Additionally, there were 4,695 proteins assigned to the Prx superfamily but not assigned to a subgroup (uncharacterized) in the SFLD.

### Active site profiling identifies protein functional site features

Active site profiling is a method used to identify the residues in the structural vicinity of a protein’s functional site (Fig 1) [36]. Briefly, key residues important for catalytic activity (Fig 1, black residues) are identified using a combination of the Catalytic Site Atlas (CSA) [64] or literature research and structure alignment. All residues within 10 Å of each key residue (Fig 1A, gray spheres) are identified and extracted from the full protein sequence and aligned N- to C- terminus to create an active site signature (Fig 1B). Fragments containing three residues or fewer are removed from the active site signature as they lack sufficient length for statistical significance. Multiple signatures are aligned to create an active site profile (ASP), characterizing the active site features of all proteins in the group (Fig 1B). An ASP score is calculated indicating the residue variation among the signatures in the profile [36]. ASP scores range from -0.5 to 1.0, where 1.0 indicates perfect alignment and conservation across all signatures.

### DASP/DASP2 utilizes ASPs to search sequence databases for proteins with similar fragments

The Deacon Active Site Profiler (DASP) is a tool that uses ASPs to search sequence databases for proteins with fragments similar to the active site motifs [37,38,55]. The ASP is separated into aligned motifs which contain contiguous fragments within the signatures (Fig 1, colored fragments). For each aligned motif, a position specific scoring matrix (PSSM) [65] is calculated, detailing the propensity for specific residues to appear in each position of the motif, normalized to the background frequency of each residue in the database [35,37,55]. Starting with the longest motif, a sliding window search is performed along each sequence in the database. A p-value defining the similarity between the ASP motif and the sequence fragment is calculated for every position; the most significant p-value indicates the best match between a fragment and the motif for a given protein. All motifs are searched in this manner to identify the best matching fragment with the caveat that fragment matches cannot overlap. For each protein sequence, the p-values for each “best matched” fragment are combined using QFAST [66] to calculate a DASP search score. This score represents the probability a given sequence contains the fragments matching the ASP motifs by chance. This process is completed across all protein sequences in the database, such that each protein is associated with a DASP search score indicating the statistical significance of the match between the protein fragments and the ASP fragments.

To efficiently perform iterative DASP searches, a new version of DASP named DASP2 was developed to support variable input formats and decrease GenBank search times. DASP2 testing demonstrated DASP and DASP2 return essentially identical data, but DASP2 searches are significantly more efficient [53]. Additionally, expanding the supported input formats allows the identified fragments of one search to be used as the input of the next search, opening the door for iterative database searches used in the MISST process. While these changes do not alter the search results, the latest version supports efficient, iterative GenBank searches which are critical to this work.

### TuLIP clusters protein structures into functionally relevant groups using DASP2

Previously, Leuthaeuser and coworkers demonstrated that clusters identified using pairwise active site similarity networks often share more functional details than those identified using full sequence or full structure similarity networks [54]. Building on this, the Two Level Iterative clustering Process (TuLIP) was developed to identify functionally relevant groups of protein structures based on active site similarity. Validation was previously performed on the enolase and GST superfamilies. Results demonstrated significant correspondence to known functional groups [52].

Initially, an all-by-all network was created using the 47 non-redundant Prx structures in which each node represents one protein structure and each node pair is connected by an edge representing a pairwise ASP score. The edge threshold was incremented and the MCL clustering algorithm [67] was applied until distinct subnetworks form, such that no edges connect subnetworks to each other. At this point, an ASP is created for each subnetwork and used to search the PDB with DASP2. If the PDB search using the subnetwork’s ASP identifies only itself (the proteins within the subnetwork) at significant DASP search scores, it is defined as “functionally relevant” and removed for further analysis. For all subnetworks which are not identified as functionally relevant groups, the edge score is incremented and the process repeated. This iterative clustering process is continued until each protein is either part of a functionally relevant group or separated out as a singlet, which signifies the end of the strict clustering stage.

The full iterative approach is then repeated for the relaxed stage: a fully connected network is formed from all singlets and the edge threshold is incremented to form subnetworks which are used to search the PDB for identifying functionally relevant groups. The relaxed stage uses more relaxed parameters for evaluation of the functional relevance of each subnetwork. Again, any subnetwork that meets the relaxed parameters is defined functionally relevant and is removed. The edge threshold is then incrementally increased. Once all proteins are either members of a functionally relevant group or singlets, TuLIP is complete. Utilizing two stages of iteration allows identification of functionally relevant groups whose relationship might be obscured by the more coherent groups identified with the strict clustering parameters.

### MISST—an iterative method to agglomerate sequences and organize clusters that share similar active site features

TuLIP is performed only on proteins of known structure. A single DASP2 search can expand the group into sequence space; however, the identified sequences are limited by the diversity of the search ASP, which, in turn, is limited to those sequences represented in the structure database. To expand functionally relevant clustering, so that the diversity of sequences and functionally relevant groups are fully comprehended, the Multi-level Iterative Sequence Searching Technique (MISST) was developed (Fig 4). This process utilizes iterative DASP2 GenBank searches to populate each TuLIP group with sequences sharing active site similarity, thus increasing robustness of the search ASP. Additionally, a novel PSSM Analysis method identifies when and how a MISST group should be subdivided into distinct functionally relevant groups.

To initiate MISST, an ASP is created for each TuLIP group; each ASP is used in an initial DASP2 search, Search0, of GenBank (Fig 4A). Given the limited representation in the structure database, the active site diversity of these initial ASPs is limited; thus, the goal of Search0 is to create a more robust ASP better representing each group’s functional site diversity. A DASP2 score of ≤1e-12 was chosen as the threshold for inclusion of sequences in the more robust profile. Previous work had identified ≤1e-8 or ≤1e-10 as “generous” and “trusted” DASP score thresholds in a single search of Prx subgroups [35]. Subsequent work on the enolase superfamily demonstrated that cross-hits (sequences identified as members of more than one functional group) decreased to zero at ≤1e-13 in the 26 subgroups and families of the enolase superfamily [52]. Balancing performance, precision and recall on the enolase superfamily, a “trusted” score threshold of ≤1e-12 was identified and is therefore used here. A detailed analysis and discussion of these score thresholds is provided in Supplemental Methods in S3 File.

An ASP is created from the pseudo-signatures of sequences identified with DASP2 search scores more significant than the score threshold. To create each pseudo-signature, fragments identified in each sequence as matching each ASP motif are concatenated (in length order, longest to shortest). The pseudo-signatures are aligned to create a new ASP for each group; each ASP is then used as input into a second DASP2 search of GenBank, termed Search1 (Fig 4A). At this point, an iterative process of sequence acquisition and data analysis begins for each TuLIP group. The DASP2 score threshold for Search1 and beyond is ≤1e-14, rather than ≤1e-12 used at Search0. 1e-14 was determined to be a more appropriate threshold because the ASPs become more robust and the DASP2 scores of known true positives shift to more significant scores with the addition of new sequences at each search (S3 File, S6 Fig). Notably, there is no score shift after Search 1 as the average DASP search score for true positives does not improve between Search 1 and Search 2 or beyond (S3 File, S6 Fig).

Beginning with Search1, each group is analyzed against two self-identification criteria to determine if the group is self-contained and stable (Fig 4A). This approach to identifying functionally relevant groups is novel as groups are not identified based on a specific threshold, but instead all groups are required to pass a self-identification test to be considered functionally relevant. In this way, groups which are functionally distinct and easier to identify can be fully identified in few iterations, while groups sharing similar active site features with other groups may take more iterations to be distinctly identified. This approach prevents the simultaneous subdivision of some groups and combination of other groups that is prevalent in most clustering.

A group is complete and removed from the iterative process when a GenBank search demonstrates self-identification; that is, all inputs are identified with significant DASP2 search scores and nothing else is identified with significant DASP2 search scores, within a small range of error. Quantitatively, two metrics define the self-identification criteria: percent new hits and percent inputs hit. The first metric tracks whether the search identified sequences not identified in the previous search: if ≤15% of the sequences identified at a score threshold of ≤1e-14 are “new” (not identified ≤1e-14 in the previous search), the group meets this metric. The second metric evaluates whether the proteins used as input were identified in this search. To pass, ≥95% of input proteins must be identified at a DASP2 score threshold of ≤1e-14 (see Supplemental Methods in S3 File for more detail). A MISST group is removed from the iterative process if it meets both metrics (Fig 4A).

The values of these two parameters were chosen based on data from the Prx superfamily, but will be evaluated on other superfamilies in the future. For completed groups in the current data set, percent new hits averaged 5.4% with a range from 2.2% to 11.3% and incomplete groups averaged 63.4% with a range from 29.9% to 98.2%. Similarly, percent inputs hit averaged 99.7% with a range from 99.5% to 100% for complete groups and averaged 66.8% with a range from 50.9% to 99.3% for incomplete groups. Preliminary analysis with other SFLD superfamilies (enolase, crotonase, and radical SAM) suggests these parameters are relatively generalizable, but comprehensive testing is required on more data sets.

Once all groups meet the self-identification criteria, a final ASP is constructed from each MISST-identified group and used to search GenBank one additional time to obtain the final MISST search results for that superfamily. The ASPs of completed searches can additionally be used at any future time to identify new sequences recently added to GenBank.

At each iteration, all groups that do not pass self-identification criteria are evaluated using the following protocol (Fig 4A, gray box):

1. **Agreement criteria:** PSSM Analysis identifies the potential for two sub-clusters to be functionally distinct. If a group was subdivided by PSSM Analysis in the previous iteration, the agreement criteria is used to determine if the search results are distinct. In this step, sequences identified by DASP2 searches from a subdivided group are compared. First, shared sequences—sequences identified in both searches at any score—are identified. Quantitatively, we count the number of shared sequences in which: 1) the DASP2 search score is ≤1e-14 (labeled “significant”) in both groups; and 2) the DASP search score is >1e-14 (“not significant”) in both groups. The sum of these two values is the number of sequences identified in both searches that share the same label (significant or not significant). This value is divided by the total number of sequences in the smaller group, producing a value from 0 to 1 indicating the overall “agreement” (in both identification and significance of DASP2 score) between the search results of the two groups. If the agreement is ≥ 0.70, the search results are identified as “the same” and the smaller group is removed from further analysis. If the agreement is < 0.7, both groups are kept. Visually, this concept is illustrated in Fig 4B for one group, Group 4, which was subdivided by PSSM Analysis into groups 4A and 4B. Distinct groups would have more sequences in the graph quadrants shaded cyan; groups that are deemed similar share more sequences in the yellow quadrants.
2. **Remove cross-hits between all groups:** To create robust and well-defined ASPs for each group, cross-hits, or sequences identified by more than one search, are removed, as previously described [35]. Briefly, if a sequence is identified in two groups with DASP2 search scores within two orders of magnitude, the sequence is removed from both groups as it is not clear to which group the protein belongs. If the two DASP2 search scores are separated by more than two orders of magnitude, the sequence is removed from the group in which it was identified at a less significant score; it remains in the group in which it was identified at a more significant score. If a sequence is identified by more than two groups, the two most significant DASP search scores are used for this analysis and the protein is removed from all other groups.
3. **Reduce redundancy within each group.** An abundance of similar protein sequences in a given group can weight an ASP in a way that is not biologically relevant. Thus, within each MISST group, proteins are analyzed using CD-Hit [68,69] with a 0.95 threshold and the suggested word size of 5. All proteins are then clustered into groups based on 95% sequence identity and the default representative is taken forward.

These three steps are completed for each MISST group at each search iteration (Fig 4A). After completion of these three steps, PSSM Analysis (Fig 4C; see subsequent section) is performed to determine potential group subdivision.

### PSSM Analysis identifies if and how each MISST group should be subdivided

Position Specific Scoring Matrix (PSSM) Analysis is a novel approach using PSSMs to determine whether a group of protein sequences contains more than one identifiable functionally distinct group based on residue similarity within the active site signatures. In this way, MISST groups that contain multiple functionally-distinguishable families can be appropriately subdivided.

PSSM Analysis begins by placing every protein identified by one group’s search into order of magnitude “bins” based on the DASP2 search score at which they were identified. Each order of magnitude is considered a bin, such that proteins with DASP search scores >1e-9 and ≤1e-8 are placed into the bin labeled “8” (Fig 4Ci). All proteins with DASP search scores ≤1e-25 are placed into the bin labeled “25.” Bin-specific ASPs are created from the proteins in each bin (using the pseudo-signatures described previously) and a PSSM [65] is calculated for the each ASP, resulting in 18 bin-specific PSSMs (Fig 4Ci). The PSSM values are based on the count of each residue in each position of the profile, normalized to the overall count of that residue in the database.

To identify the similarity between proteins in each pair of bins, a modified Pearson correlation coefficient is calculated pairwise between bin-specific PSSMs. A PSSM is a two-dimensional array, the first dimension representing each of the 20 amino acids; the second dimension representing a position in an ASP (positions in an ASP are indicated by arrows in Fig 1B). The standard Pearson correlation coefficient is calculated between analogous columns of a pair of PSSMs. To get the overall comparison between two PSSMs, column correlations must be summarized, but averaging correlation coefficients can lead to bias [70]. Therefore, a Fisher transformation is executed prior to computing the average. Due to the nature of the transformation, all coefficients >0.9999 are set equal to 0.9999, and the Fisher transform is performed to produce a z-score for each column. The z-scores are then averaged across all columns and back transformed to r, producing the modified Pearson correlation coefficient, which correlates the active site similarity between the proteins in two bins.

To define when a group should be subdivided, a fully connected network is created, with each node representing proteins in a scoring bin (from 8 to 25) and each edge representing the pairwise correlation coefficient between bin-specific PSSMs (Fig 4Cii). Beginning at the lowest correlation value (rounded to two decimal places), a filter threshold is applied to the network, removing all edges below the threshold. The filtered network is clustered using MCL clustering [67] to produce subnetworks (Fig 4Ciii). If distinct subnetworks are formed, in which no edges connect the two (or more) subnetworks to each other, the subnetworks are evaluated based on the following criteria to determine if they might represent functionally distinct groups: 1) each subnetwork must contain at least three nodes; and 2) the nodes (bins) must represent contiguous DASP2 scores (e.g. 8, 9, and 10 rather than 8, 10, and 12). If the subnetworks meet both criteria, the subnetwork containing the nodes with the least significant DASP2 scores is removed as a potential functionally relevant group, while the remaining subnetwork is subdivided further. If a subnetwork does not meet both criteria, it is not identified as a potential functionally distinct cluster. The filter threshold is increased by 0.02 each iteration and the clustering process is repeated.

At the edge threshold of 0.98, PSSM Analysis is completed. If a group has subdivided, ASPs are built from the pseudo-signatures of proteins in each subnetwork and used in the subsequent MISST iteration and search of GenBank (Fig 4A). If the network reaches the 0.98 edge threshold and no subnetworks have been identified, an ASP is created from the pseudo-signatures of the sequences with DASP2 search scores ≤1e-14.

### Quantitative analysis of final MISST groups

MISST iterations continue, as outlined in Fig 4. Once all groups pass self-identification criteria, a final DASP2 search of GenBank is completed for each MISST-identified group. In this work, these final searches were completed in March 2016. Cross hit analysis then identifies the number of shared sequences between the six groups identified at the significance threshold of ≤1e-14. Cross-hits are identified and removed using the same procedure utilized during the MISST process (Fig 4A). The final list of all proteins identified in each MISST group along with their DASP2 search score, SFLD annotation, and pseudo-signature can be found in S2 File.

The results of these searches were compared to the expert-identified subgroups using quantitative methods previously used to evaluate other similar processes [49,50,59,62]. To calculate these measures, the MISST groups were compared to the sequences in the SFLD as of March 6^th^, 2016 (http://sfld.rbvi.ucsf.edu/django/). Each of the 6 MISST groups contained the majority of one subgroup; consequently, the analysis was completed using a 1-to-1 correspondence of MISST group to known functional groups (defined in Table 1).

To evaluate how well our clusters compared with known functional clusters, measurements of purity, edit distance, and VI distance were performed, as previously described [50]. Additionally, the combined performance metric suggested by Orengo and colleagues [49] was calculated as well as the F-measure, which is the harmonic mean of precision and recall [59]. Details of these metrics are provided in S3 File.

The consensus Prx motifs for each group were determined based on the conservation of residues in each position of the motif according to the following rules: 1) if the three most conserved residues make up ≤97% of that position, an x is used in the consensus sequence for that position, and 2) for all other positions, all residues identified in ≥3% of the MISST group sequences are annotated in the consensus sequence. Conservation graphs were built using Weblogo [61].

### MISST network creation

A representative network (RepNet) was created for all 38,739 sequences identified by the six MISST groups in the final searches using Cytoscape [63]. Using CD-Hit [68,69], 1,369 clusters were identified where all members share 55% sequence identity with the representative protein. Each representative is a node in the RepNet and the edges connecting the nodes are pairwise BLAST scores between each pair of representatives. The nodes are colored by the MISST group the proteins were identified by.

## Supporting Information

S1 Fig Hierarchical clustering based on the Prx motif identifies the same groups as TuLIP. (A) The Prx motif, **P**xxx(**T/S**)xx**C**_**P**_ [3,56,57] is aligned for all 47 structural representatives from the Prx superfamily using MAFFT [71,72] and residues are colored using the Taylor coloring scheme [73] for physiochemical properties. PDBIDs are colored based on SFLD functional annotation. (B) An Average Distance tree was created using Jalview [74] applet PAM 250. Lines are colored based on SFLD functional annotation, and the red vertical line indicates the separation of clusters most similar to known SFLD functional groups.
(TIF)

S2 Fig Structures show conserved residues near the active site for each of the six MISST-identified Prx groups. A representative structure with PDBID is shown for each of the six final MISST groups. Active site fragments are colored based on brace colors in Fig 5. Highly conserved residues in each subgroup are shown with the ball and stick representation. The C_P_ is shown in the spherical representation and colored lime green. Gray side chains represent the conserved Arg in position 36 of the signature conservation logos (Fig 5). Brown side chains represent the Prx motif. Black side chains represent residues conserved within the subgroup that may be of interest. Side chains of other colors (cyan, yellow, and light pink) are specifically discussed in the text. Molecular visualizations were created with UCSF Chimera package, version 1.10.2 [75].
(TIF)

S3 Fig Heat maps show the phylogenetic distribution of residues in select positions of the active site profiles. For each of the six MISST groups, the percent of the group classified in each phylogenetic class is shown as a heat map using red tones (see legend). Phylogenetic classifications which comprise ≤2% of any given MISST group are not shown. As 99% of the Sct2_Tpx MISST group is bacterial sequences, the heat map is not included. For each phylogenetic classification, the percent of sequences with different residues at selected positions of the active site signature is shown as a heat map using blue tones (see legend). The signature position (corresponding to the signature conservation graphs in Fig 5) is shown across the top of the heat map. Only select positions which contain two or three main residues are shown. Colored brackets are discussed in the text.
(TIF)

S4 Fig Phylogenetic distributions of MISST-identified Prx groups and Sct4_Prx1 GG(L/I)G motif. (A) Pie charts illustrate phylogenetic distribution of each of the six MISST-identified Prx groups. (B) The phylogenetic distribution of proteins identified in the Sct4_Prx1 MISST group with the GGLG and GGIG motif is shown as pie charts (top). The distribution of all bacteria identified in the Sct4_Prx1 MISST group and the bacteria identified with the GGIG motif are displayed as pie charts (bottom), where fill color represents the phylum classification for each bacterial protein. All proteins identified by MISST at the final DASP score threshold ≤1e-14 (after final cross-hit analysis) were used in these analyses.
(TIF)

S5 Fig Network clusters at four DASP2 search score thresholds demonstrate the discreteness of the six MISST groups. Networks were created for the final MISST searches with significance thresholds ≤1e-8 (A), ≤1e-10 (B), ≤1e-12 (C), and ≤1e-14 (D); each protein is a node and each edge represents a DASP2 search score connecting the protein to the MISST search. The networks were created prior to the final cross hit analysis of the completed, self-identified groups. Nodes are colored based on SFLD annotations as shown in legend. The networks were created using Cytoscape with the force directed layout [63].
(TIF)

S6 Fig Change in true positive DASP search scores between MISST iterative searches. The magnitude change in DASP search score for true positive proteins identified in successive iterative DASP searches is shown as a series of boxplots corresponding to iterative searches 0 to 3.
(TIF)

S7 Fig Histograms for each final Prx MISST DASP search showing input classification. For each of the six MISST isofunctional groups, the final DASP search results are displayed as a histogram with each protein colored based on whether or not it was an input to the search (or 95% identical to an input). The dashed line represents the trusted significance threshold ≤1e-14 used for quantitative analysis.
(TIF)

S8 Fig ROC plot for the MISST-identified Prx isofunctional groups. For each isofunctional group, the final search data was categorized into input and not input. These categories were used to define TP, FP, TN, and FN which were then used to build an ROC plot for each group using thresholds ≤1e-8 to ≤1e-30. The combined data for all six groups is shown in black. For each curve, the trusted significance threshold ≤1e-14 is indicated with a large box symbol with white fill.
(TIF)

S1 Table All cross-hits identified at the significance threshold ≤1e-14. Scores in bold indicate the protein was removed from that group (see Methods).
(DOCX)

S1 File A text file containing the active site profiles used for the initial DASP2 searches of GenBank. (TXT)

S2 File An Excel file containing all sequences identified with DASP2 search scores ≤1e-14 in the final MISST search following cross-hit analysis. One worksheet is provided for each of the six MISST-identified groups. GI number, GenBank accession number, DASP2 search score, pseudo-signature, and SFLD annotation are provided.
(XLSX)

S3 File A Word file containing supporting results discussing the phylogenetic distribution of residues in the active site profiles of the six MISST-identified isofunctional groups, and supporting methods describing the DASP search score threshold and it’s generalizability, the self-identification criteria used for MISST, and the quantitative analysis performed on the final MISST groups, including F-measure and performance analysis. (DOCX)
