# Supplementary figures and images for: An Atlas of Peroxiredoxins Created Using an Active Site Profile-Based Approach to Functionally Relevant Clustering of Proteins
Source: PLoS Comput Biol. 2017 Feb 10;13(2):e1005284. doi: 10.1371/journal.pcbi.1005284 (PMC5302317; doi:10.1371/journal.pcbi.1005284)

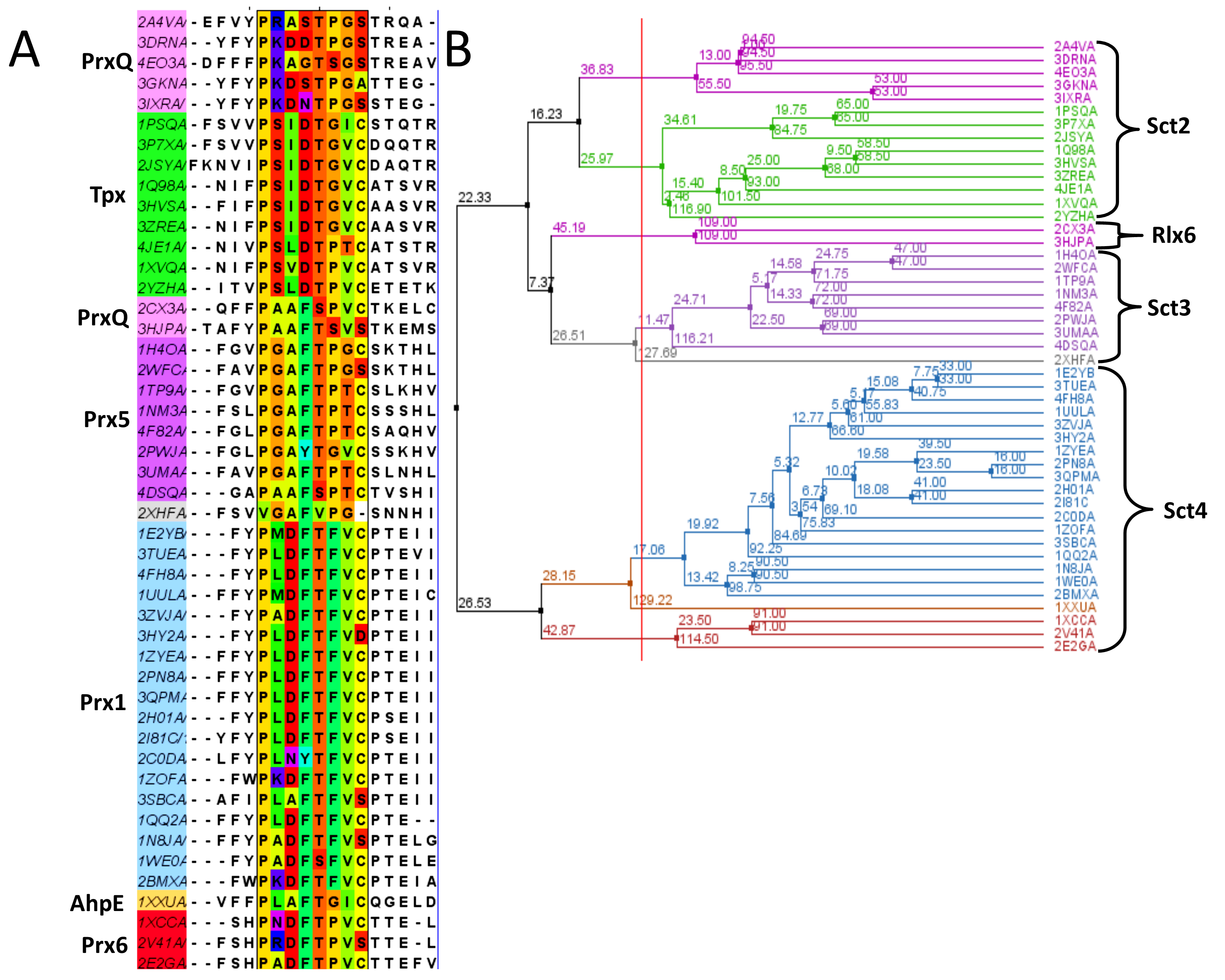

Supplement: S1 Fig — (A) The Prx motif, Pxxx(T/S)xxCP [3,56,57] is aligned for all 47 structural representatives from the Prx superfamily using MAFFT [71,72] and residues are colored using the Taylor coloring scheme [73] for physiochemical properties. PDBIDs are colored based on SFLD functional annotation. (B) An Average Distance tree was created using Jalview [74] applet PAM 250. Lines are colored based on SFLD functional annotation, and the red vertical line indicates the separation of clusters most similar to known SFLD functional groups. (TIF) [file pcbi.1005284.s001.tif]

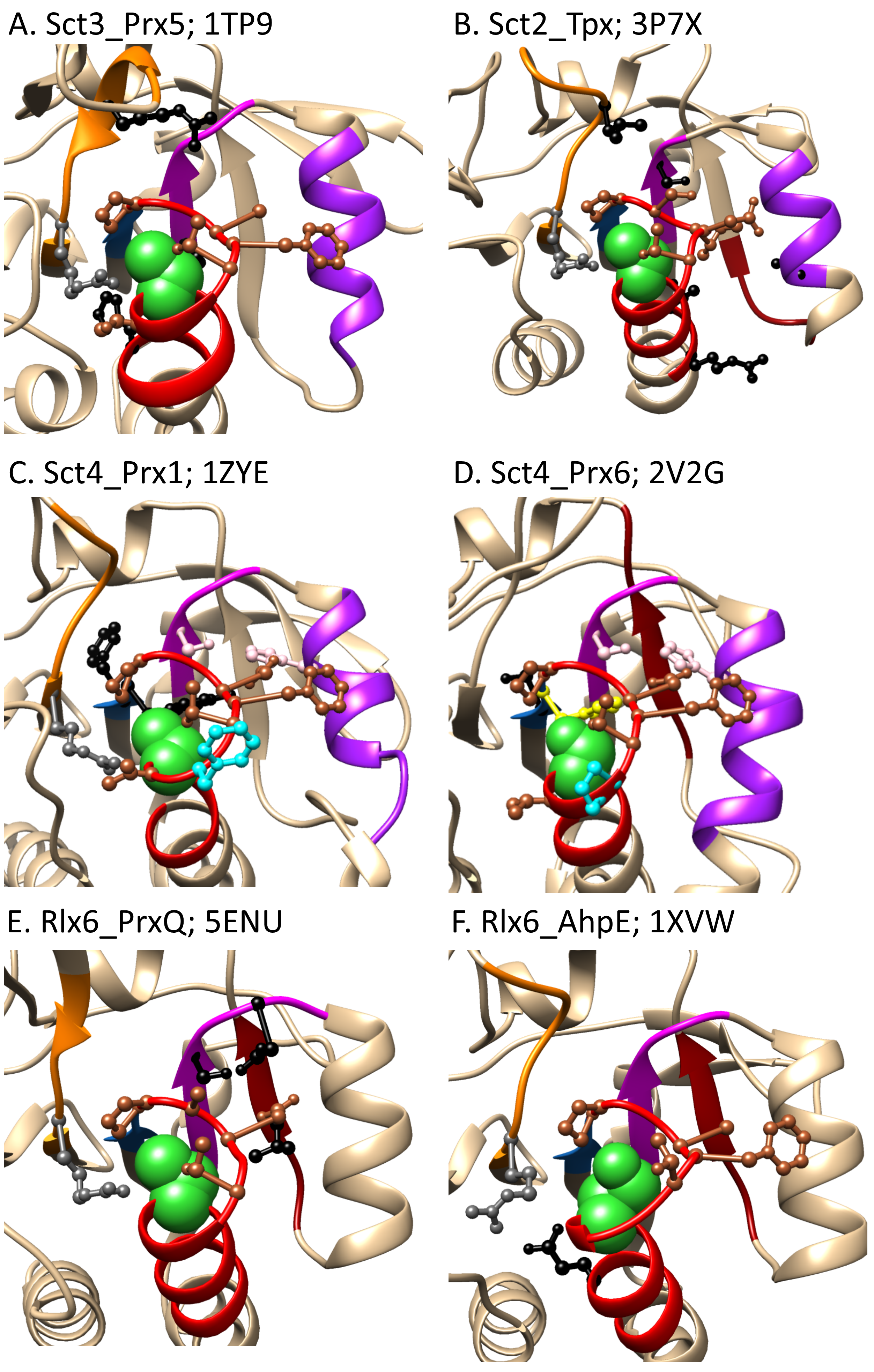

Supplement: S2 Fig — A representative structure with PDBID is shown for each of the six final MISST groups. Active site fragments are colored based on brace colors in Fig 5. Highly conserved residues in each subgroup are shown with the ball and stick representation. The CP is shown in the spherical representation and colored lime green. Gray side chains represent the conserved Arg in position 36 of the signature conservation logos (Fig 5). Brown side chains represent the Prx motif. Black side chains represent residues conserved within the subgroup that may be of interest. Side chains of other colors (cyan, yellow, and light pink) are specifically discussed in the text. Molecular visualizations were created with UCSF Chimera package, version 1.10.2 [75]. (TIF) [file pcbi.1005284.s002.tif]

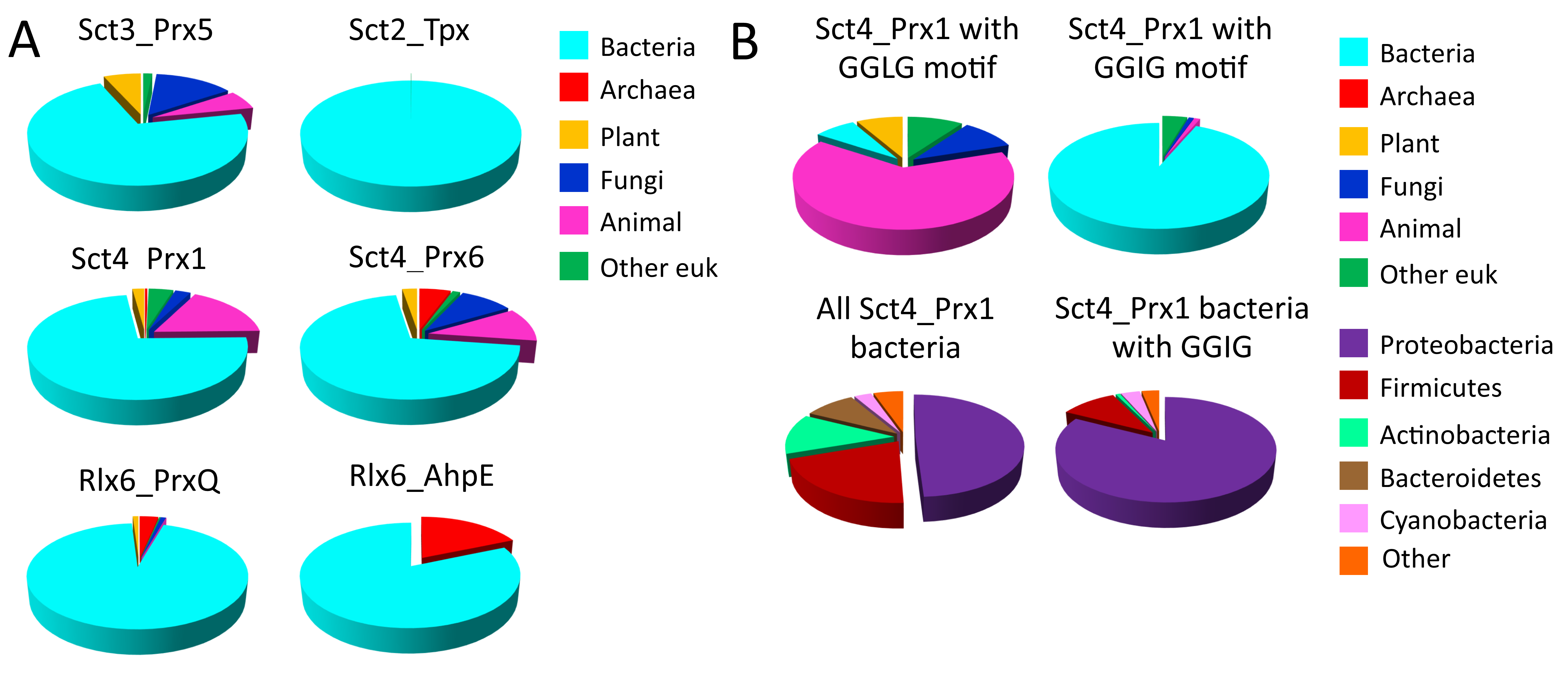

Supplement: S3 Fig — For each of the six MISST groups, the percent of the group classified in each phylogenetic class is shown as a heat map using red tones (see legend). Phylogenetic classifications which comprise ≤2% of any given MISST group are not shown. As 99% of the Sct2_Tpx MISST group is bacterial sequences, the heat map is not included. For each phylogenetic classification, the percent of sequences with different residues at selected positions of the active site signature is shown as a heat map using blue tones (see legend). The signature position (corresponding to the signature conservation graphs in Fig 5) is shown across the top of the heat map. Only select positions which contain two or three main residues are shown. Colored brackets are discussed in the text. (TIF) [file pcbi.1005284.s003.tif]

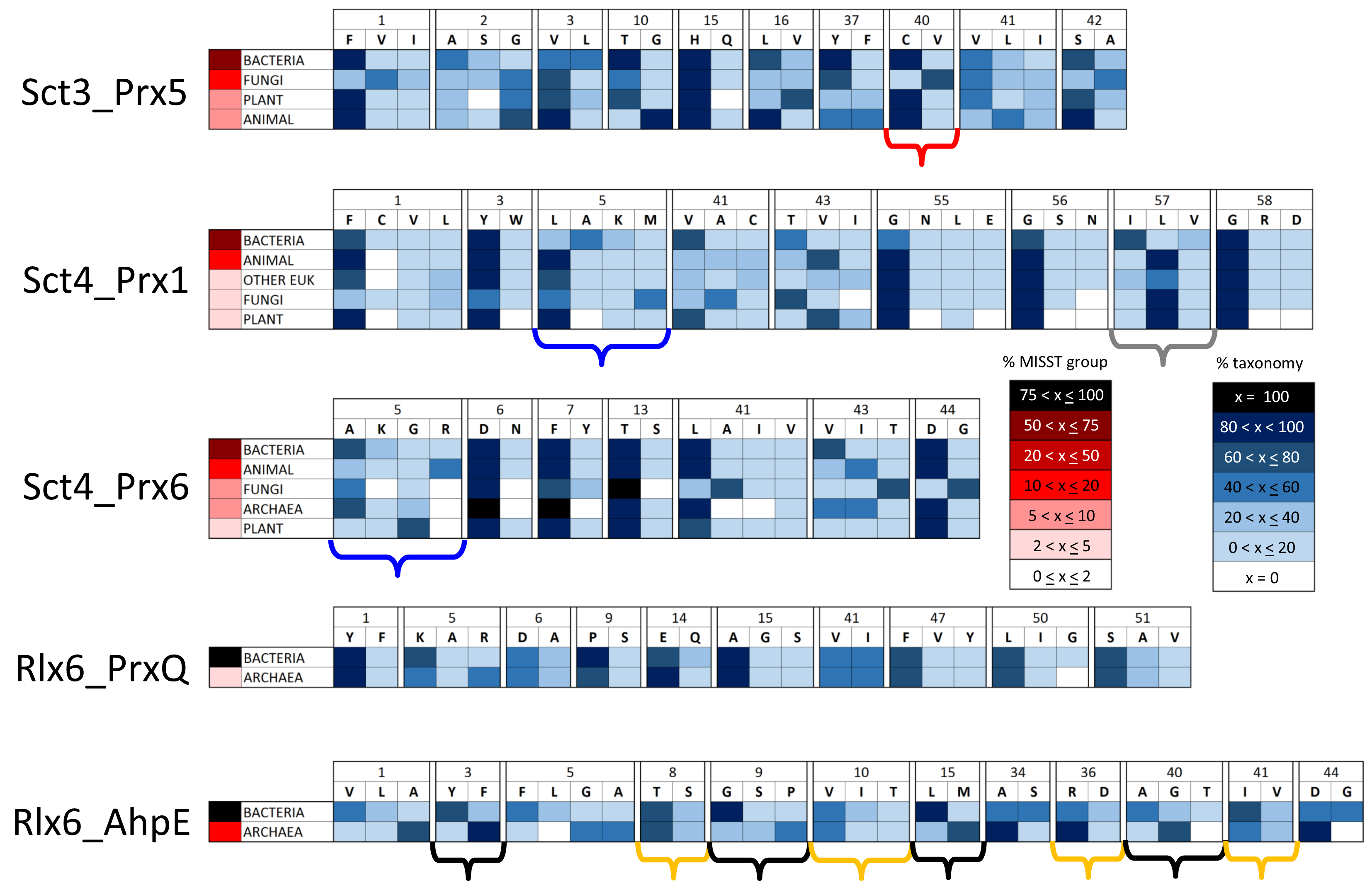

Supplement: S4 Fig — (A) Pie charts illustrate phylogenetic distribution of each of the six MISST-identified Prx groups. (B) The phylogenetic distribution of proteins identified in the Sct4_Prx1 MISST group with the GGLG and GGIG motif is shown as pie charts (top). The distribution of all bacteria identified in the Sct4_Prx1 MISST group and the bacteria identified with the GGIG motif are displayed as pie charts (bottom), where fill color represents the phylum classification for each bacterial protein. All proteins identified by MISST at the final DASP score threshold ≤1e-14 (after final cross-hit analysis) were used in these analyses. (TIF) [file pcbi.1005284.s004.tif]

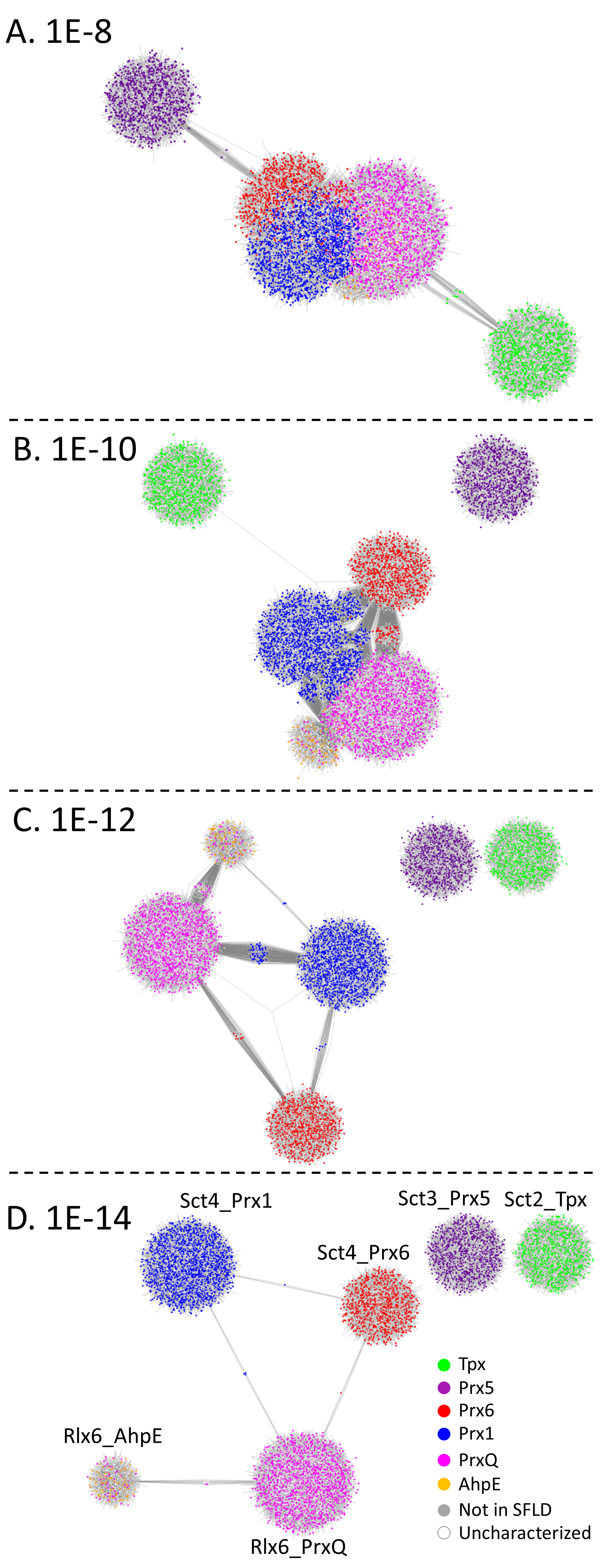

Supplement: S5 Fig — Networks were created for the final MISST searches with significance thresholds ≤1e-8 (A), ≤1e-10 (B), ≤1e-12 (C), and ≤1e-14 (D); each protein is a node and each edge represents a DASP2 search score connecting the protein to the MISST search. The networks were created prior to the final cross hit analysis of the completed, self-identified groups. Nodes are colored based on SFLD annotations as shown in legend. The networks were created using Cytoscape with the force directed layout [63]. (TIF) [file pcbi.1005284.s005.tif]

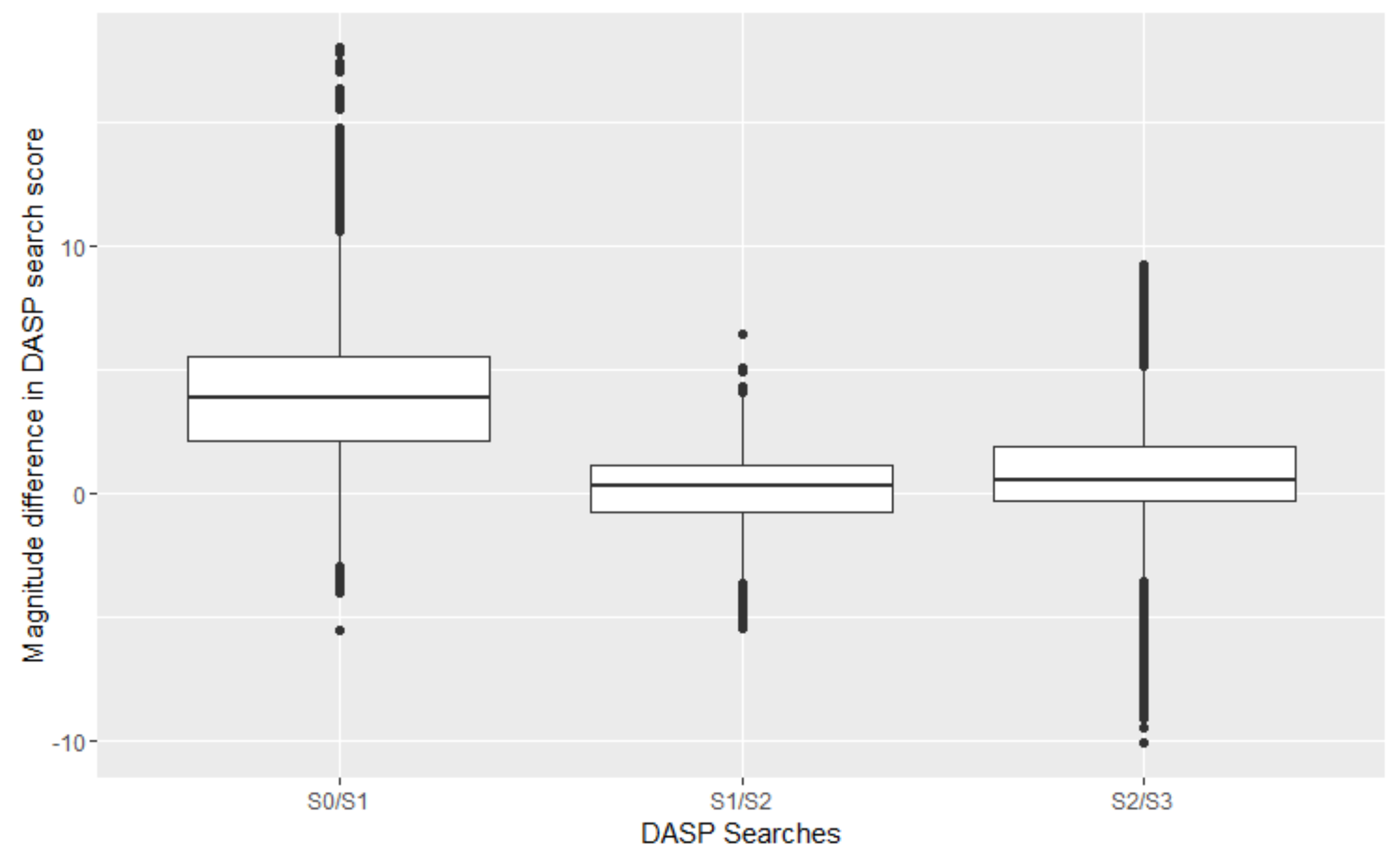

Supplement: S6 Fig — The magnitude change in DASP search score for true positive proteins identified in successive iterative DASP searches is shown as a series of boxplots corresponding to iterative searches 0 to 3. (TIF) [file pcbi.1005284.s006.tif]

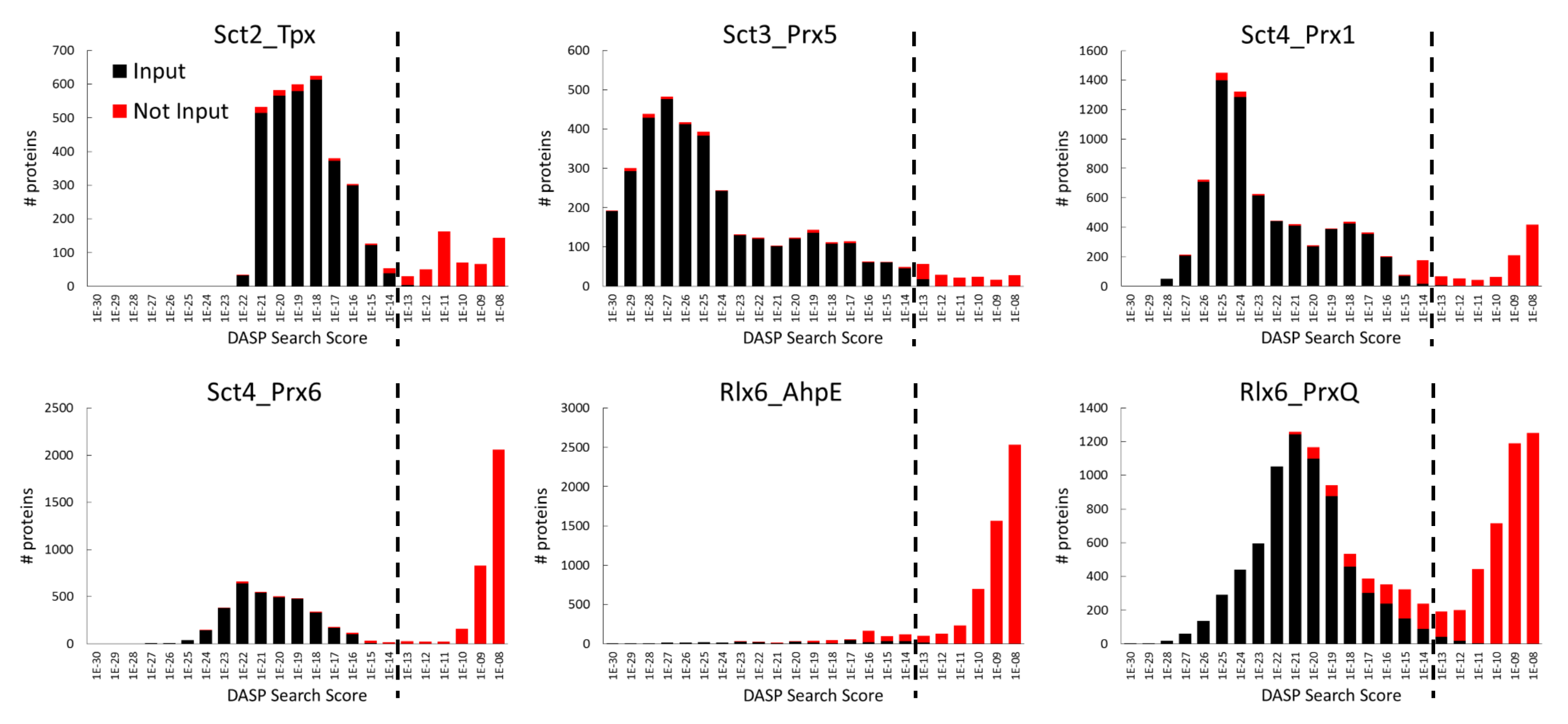

Supplement: S7 Fig — For each of the six MISST isofunctional groups, the final DASP search results are displayed as a histogram with each protein colored based on whether or not it was an input to the search (or 95% identical to an input). The dashed line represents the trusted significance threshold ≤1e-14 used for quantitative analysis. (TIF) [file pcbi.1005284.s007.tif]

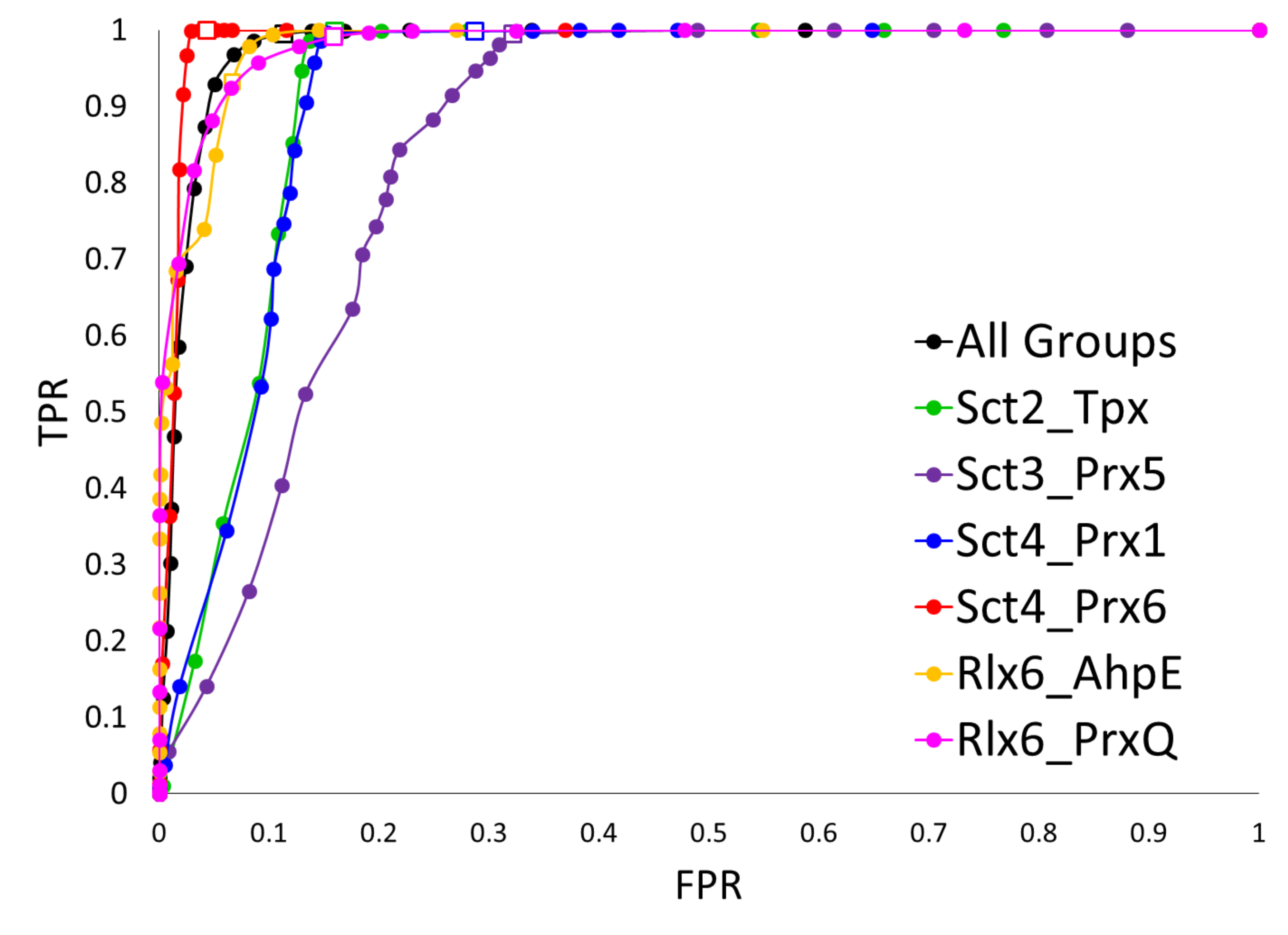

Supplement: S8 Fig — For each isofunctional group, the final search data was categorized into input and not input. These categories were used to define TP, FP, TN, and FN which were then used to build an ROC plot for each group using thresholds ≤1e-8 to ≤1e-30. The combined data for all six groups is shown in black. For each curve, the trusted significance threshold ≤1e-14 is indicated with a large box symbol with white fill. (TIF) [file pcbi.1005284.s008.tif]
