# Supplementary material for: An Atlas of Peroxiredoxins Created Using an Active Site Profile-Based Approach to Functionally Relevant Clustering of Proteins
Source: PLoS Comput Biol. 2017 Feb 10;13(2):e1005284. doi: 10.1371/journal.pcbi.1005284 (PMC5302317; doi:10.1371/journal.pcbi.1005284)
Supplement: S1 Table — Scores in bold indicate the protein was removed from that group (see Methods). (DOCX) [file pcbi.1005284.s009.docx]

| Accession/Version | Sct2_Tpx Score | Sct3_Prx5 Score | Sct4_Prx1 Score | Sct4_Prx6 Score | Rlx6_AhpE Score | Rlx6_PrxQ Score |
| --- | --- | --- | --- | --- | --- | --- |
| WP_048641542.1 |  |  | 2.37E-21 | **2.26E-15** |  |  |
| WP_014019877.1 |  |  | 5.00E-21 | **3.45E-15** |  |  |
| WP_020889726.1 |  |  | 4.31E-20 | **5.65E-15** |  |  |
| AEW04094.1 |  |  |  | 8.33E-21 |  | **6.52E-15** |
| AEJ40302.1 |  |  |  | 8.59E-21 |  | **5.28E-15** |
| CDA17095.1 |  |  |  | 2.85E-25 |  | **5.29E-15** |
| CDE10030.1 |  |  |  | 3.01E-23 |  | **6.46E-15** |
| XP_002140963.1 |  |  | 6.81E-21 |  |  | **9.68E-15** |
| XP_002788581.1 |  |  | 1.80E-20 |  |  | **6.59E-15** |
| XP_002788579.1 |  |  | 2.07E-20 |  |  | **7.47E-15** |
| WP_013930131.1 |  |  |  |  | 1.24E-21 | **8.20E-15** |
| WP_056125303.1 |  |  |  |  | 5.22E-20 | **6.40E-15** |
| WP_047754200.1 |  |  |  |  | 5.67E-18 | **6.41E-15** |
| KER09617.1 |  |  |  |  | **1.27E-15** | **1.80E-17** |
| WP_012466961.1 |  |  |  |  | **4.88E-16** | **7.89E-16** |
| WP_059139175.1 |  |  |  |  | **4.89E-16** | **2.80E-15** |
| ADB37633.1 |  |  |  |  | 1.67E-23 | **1.19E-15** |
| WP_026416828.1 |  |  |  |  | 2.95E-23 | **4.95E-16** |
| WP_008509451.1 |  |  |  |  | 2.93E-22 | **1.53E-15** |
| WP_026903835.1 |  |  |  |  | 3.00E-22 | **8.41E-15** |
