# Supplementary material for: An Atlas of Peroxiredoxins Created Using an Active Site Profile-Based Approach to Functionally Relevant Clustering of Proteins
Source: PLoS Comput Biol. 2017 Feb 10;13(2):e1005284. doi: 10.1371/journal.pcbi.1005284 (PMC5302317; doi:10.1371/journal.pcbi.1005284)
Supplement: S3 File — (DOCX) [file pcbi.1005284.s012.docx]

**Supplemental Results**

**Phylogenetic analysis reveals intriguing results for four MISST groups**

The majority of Sct3_Prx5 proteins are found in bacteria (Fig. S4A), similar to that previously reported on a significantly smaller dataset (Nelson et al., Proteins 2011). Phylogenetic analysis indicates that the Cys of the (C/V)(V/L/I)(S/A)VN fragment (Fig. 5A, fuchsia brace) is found in most bacteria, animal, and plant sequences, while Val is found in most fungal sequences (Fig. S3, red bracket). This residue is located in helix α5 and, when a cysteine, sometimes serves as the C_R_ (Perkins et al., Trends Biochem Science 2015). In the fungal sequences, which do not contain a Cys in helix α5, a conserved Cys is identified in the loop between β1 and β2. Previous work demonstrated the C_R_ in Prx5 proteins is in this location in 17% of the subgroup. Altogether, this suggests the location of the C_R_ in the Prx subgroup may be segregated by phylogenetic classification.

Evaluation of phylogenetic distribution shows that Sct4_Prx1 and Sct4_Prx6 are 73.2% and 70.1% found in bacteria, respectively (Fig. S4A), representing larger percentages of bacterial proteins than previously determined on a much smaller dataset (Poole et al., Mol Cells 2016). Phylogenetic distribution of residues at the active site can also be observed. For instance, signature position 5 is not particularly conserved in either of these groups (Fig. S3, blue brace), but in Prx1, this residue is Leu in most organisms and Ala in bacteria. Conversely, in Prx6, this residue is Ala in bacteria, fungi and archaea, Arg in animals, and Gly in plants. Signature position 41 also shows distinctive phylogenetic distribution (Fig S3).

The distinctive GG(L/I/V)G motif previously identified (Wood et al., Science 2003) is observed in the Prx1 sequence signatures (Fig. 5C, gray brace), but not in other subgroups. This signature distinguishes “sensitive’’ Prxs which are more prone to C_P_ over-oxidation than the more “robust” bacterial Prxs (Wood et al., Science 2003). Subsequent analysis identified the GGIG variant of this motif in some pathogenic bacteria (Hall et al., FEBS 2009). In our own analysis, this residue shows the same phylogenetic distribution (Fig S3, gray brace). Of the 2386 Sct4_Prx1 proteins with a GGLG motif, 7.4% are bacteria and 92.5% are eukaryote. Of the 1978 proteins with a GGIG motif, 93.6% are bacteria and 6.4% are eukaryote. Detailed evaluation of the bacterial Sct4_Prx1 proteins indicates only 49.2% are proteobacteria, while 82.6% of the bacterial proteins containing the GGIG motif are proteobacteria (Fig. S4B). This more complete analysis supports previous findings that the GGIG motif is often seen in pathogenic bacteria (Hall et al., FEBS 2009).

All the AhpE subgroup members previously identified (Nelson et al., Proteins 2011) are bacterial proteins from the phylum actinobacteria (Poole et al., Mol Cells 2016), while the Rlx6_AhpE MISST group is composed of 81% bacteria and 19% archaea (Fig. S4A). Of the 98 sequences previously annotated to AhpE and identified as part of Rlx6_AhpE, all are bacterial sequences with 95 in the phylum actinobacteria and 3 in the phylum verrucomicrobia. Of the 54 Rlx6_AhpE sequences previously annotated as PrxQ, 37 are archaea (euryarchaeota, crenarchaeota, and thaumarchaeota phyla) and 17 are bacteria (seven phyla, but no actinobacteria). Phylogenetic distribution of residue identity shows some positions are conserved in both bacterial and archaeal proteins (Fig. S3, gold brackets). At other positions, residue identity in proteins of bacterial and archaeal origin is distinct (Fig. S3, black brackets). Clearly, there are characteristics that distinguish bacterial and archaeal proteins in the Rlx6_AhpE subgroup. Experimental analysis is required to better understand commonalities and differences between the molecular functional details.

**Supplemental Methods**

**Detailed analysis of DASP search score thresholds**

Initial DASP searches using the expert-defined Prx subgroups were completed by Nelson et al. and a trusted significance threshold of ≤1e-10 was determined for distinctly identifying proteins in the proper subgroup search (Nelson et al., Proteins 2011). As MISST is an iterative process aimed to gather proteins in each isofunctional group starting from structural representatives, the initial search (Search0) utilizes small input sets. To create more robust ASPs which drive the iterative process, the initial score threshold must be more stringent to avoid proteins being identified in multiple groups. Therefore, the trusted score threshold for Search0 is ≤1e-12.

As the ASPs become more robust going into Search1, the DASP search scores for true positive proteins are ~4 orders of magnitude more significant (Fig. S6). However, with the more robust profiles, the search score can be relaxed again to better encapsulate all proteins in the isofunctional group. Therefore, the trusted score threshold for Search1 is ≤1e-14, or 4 orders of magnitude more significant than the Search0 threshold for single DASP searches and 2 orders of magnitude more significant than the Search0 threshold for iterative DASP searches. Going forward, the search score of true positives is not significantly changed after Search1 (Fig. S6), so the trusted DASP search score threshold remains ≤1e-14 for all iterations.

To assess the generalizability of the trusted score threshold, the data from the final DASP2 search for each of the six MISST groups was evaluated to determine the ideal threshold for self-identification, where the search identifies all search inputs and no other proteins. Histograms showing the distribution of DASP search scores colored by input (red) and non-input (black) demonstrate the striking demarcation of inputs and non-inputs near the ≤1e-14 trusted threshold (dashed line) in most groups (Fig. S7). Additionally, ROC curves were created by identifying all inputs identified better than the threshold as true positives, all non-inputs identified better than the threshold as false positives, all inputs identified worse than the threshold as false negatives, and all non-inputs identified worse than the threshold (but still ≤1e-8) as true negatives (Fig. S8). The trusted threshold (squares with no fill) demonstrates a high TPR and a low FPR in all six groups. Together, these data suggest the incredible generalizability of the trusted score threshold throughout the Prx superfamily.

Validation using other known superfamilies is crucial to the definition of a generalizable, trusted significance threshold. An initial DASP search (Search0) with 26 TuLIP groups from the enolase superfamily supports the thresholds identified here (Knutson et al., BMC Bioinformatics submitted July 2016). In that work, the threshold ≤1e-12 results in just three cross-hits, or proteins identified in more than one group; thus, this same threshold was utilized for quantitative analysis of the enolase superfamily. Further, preliminary data from the radical SAM superfamily suggest the threshold ≤1e-12 for Search0 and ≤1e-14 for successive searches is an appropriate significance threshold to reduce cross-hits and improve coverage (data not shown). While more validation will be completed in the future, current data suggests a generalizable DASP search score threshold is appropriate within the Prx superfamily and throughout other SFLD superfamilies.

**Self-identification criteria: evaluating a MISST-identified group as completed**

After every iterative search, beginning with Search1, each MISST group is evaluated based on two self-identification criteria to determine if the group identified itself and nothing else in the most recent DASP2 GenBank search. First, input sequences used to create the search profile are compared to the sequences identified in the GenBank search at a DASP2 search score threshold of ≤1e-14. Percent inputs hit is calculated by:

$$Percent Inputs Hit =\frac{count of inputs identified \leq1e^{-14}}{count of all inputs}*100$$

(1)

If the percent inputs hit is ≥ 95%, the number of new hits is then evaluated. For this calculation, the percentage of sequences identified at significant scores (≤1e-14) which were not identified at significant scores in the previous search is calculated, where N is the current search number.

$$Percent New Hits=\frac{count of newly sig. in SearchN}{count of all sig in SearchN}*100$$

(2)

If the percent new hits is ≤ 15%, then a MISST group passes both self-identification criteria and is removed from subsequent MISST analysis.

**Metrics for evaluation of MISST: F-Measure and performance analysis**

For the quantitative analysis, only proteins annotated to one of the six Prx subgroups are included in the calculations because a gold standard set is required for comparison. All proteins identified by MISST that are not in the SFLD or are uncharacterized in the SFLD cannot be compared to known annotations. Additionally, F-measure was calculated both pre-cross hit analysis and post-cross hit analysis (see Methods for details). Performance requires all proteins to be in just one group so this analysis was only completed post-cross hit analysis.

Quantitative metrics were calculated at each DASP2 search score threshold from <1e-08 to <1e-25. Each MISST-identified sequence was evaluated as a true or false positive or negative at each threshold based on the following criteria:

- True positive: a MISST-identified protein annotated to the SFLD subgroup represented by that MISST group
- True negative: a protein annotated to any SFLD Prx subgroup not represented by that MISST group and also not identified in the corresponding MISST group with a DASP2 search score more significant than the threshold being analyzed
- False positive: a MISST-identified protein annotated to any SFLD Prx subgroup that is not represented by that MISST group
- False negative: a protein annotated to the SFLD subgroup represented by that MISST group but not identified in the corresponding MISST group with a DASP2 search score more significant than the threshold being analyzed

F-measure is calculated as the harmonic mean of precision and recall. Precision and recall values are calculated using the counts of true positives, false positives, true negatives, and false negatives. Using these counts, the equations for precision, recall, and F-measure at each score threshold are given as follows:

$$Precision=\frac{TP}{TP+FP}$$

(3)

$$Recall=\frac{TP}{TP+FN}$$

(4)

$$F-measure=\frac{2*Precision*Recall}{Precision+Recall}$$

(5)

The performance metric is a combination of purity, VI distance, and edit distance, as previously described (Lee et al., Nucleic Acids Res 2010; Knutson et al., BMC Bioinformatics submitted July 2016). Purity is a measurement of the percentage of MISST groups that only contain proteins annotated to one subgroup. Thus, 100% purity indicates that all MISST groups are completely “pure” and only contain proteins annotated to a single subgroup. Purity is calculated as follows:

$p=$ $100*\sum_{k=1}^{K} \frac{u_{k}}{e_{k}}$ (6)

The edit distance indicates the number of changes required to transform the identified MISST groups into the six expertly curated Prx subgroups. Edit distance is calculated as follows:

$$e=2\left( \sum_{k,k^{'}} r_{k,k^{'}} \right)-K-K^{'}$$

(7)

where $r_{k,k^{'}}$ is 1 if the MISST group (k) and expert annotation (k’) share common proteins, and zero otherwise. K is the number of MISST groups (six), and K’ is the number of expert annotated groups (also six).

VI distance measures the amount of information not shared between MISST groups as a function of the information entropy H(S) and H(S’) and the combined information between the groups I(S,S’). The probability P(K) of a sequence being in any cluster is given by

$$P\left( K \right)=\frac{n_{K}}{N}$$

(8)

where $n_{K}$ is the number of sequences in the MISST group that are annotated to the expected subgroup, and $N$ is the total number of sequences annotated to that subgroup. Thus, the information entropy is given by

$$H\left( S \right)=-\sum_{k=1}^{K} P\left( k \right)logP\left( k \right)$$

(9)

where S is the MISST group with K proteins, and S’ is the annotated group with K’ proteins. The information shared between the two is

$$I\left( S,S^{'} \right)=\sum_{k=1}^{K} \sum_{k^{'}=1}^{K} P\left( k,k^{'} \right)log\left( \frac{P\left( k,k^{'} \right)}{P\left( k \right)P^{'}\left( k^{'} \right)} \right)$$

(10)

With the values of information entropy and shared information, VI can be calculated as follows:

$v=$ $H\left( S \right)+H\left( S’ \right)-2*I\left( S,S’ \right)$ (11)

Finally, performance combines these three metrics as follows:

$$Perf=\frac{2p+\left( 100-c_{e}.e \right)+\left( 100-c_{v}.v \right)}{4}$$

(12)

where p is the purity expressed as a percentage, and $c_{e}$and $c_{v}$ are scaling factors of the initial edit ($e)$ and VI distance (v) which assumes all proteins are placed into their own group (Lee *et al.*, 2010).

$$c_{e}=\frac{100}{e_{0}},c_{v}=\frac{100}{v_{0}}$$

(13)
